# Social determinants of prostate cancer in the Caribbean: a systematic review and meta-analysis

**DOI:** 10.1186/s12889-018-5696-y

**Published:** 2018-07-20

**Authors:** Catherine R. Brown, Ian Hambleton, Shawn M. Hercules, Nigel Unwin, Madhuvanti M. Murphy, E. Nigel Harris, Rainford Wilks, Marlene MacLeish, Louis Sullivan, Natasha Sobers-Grannum, The U.S. Caribbean Alliance for Health Disparities Research Group (USCAHDR), Miriam Alvarado, Nadia Bennett, Aurelian Bidulescu, Catherine R. Brown, Trevor Ferguson, Damian Francis, Ian R. Hambleton, Eon Nigel Harris, Christopher Hassell, Anselm J. M. Hennis, Shawn M. Hercules, Christina Howitt, Marlene MacLeish, Madhuvanti M. Murphy, Thelma Alafia Samuels, Natasha Sobers-Grannum, Louis Sullivan, Nigel Unwin, Rainford Wilks, Lynda Williams, Novie Younger-Coleman

**Affiliations:** 1 George Alleyne Chronic Disease Research Centre, Caribbean Institute for Health Research, The University of the West Indies, Bridgetown, Barbados; 2 Department of Biology, McMaster University, Hamilton, ON Canada; 3 MRC Epidemiology Unit, University of Cambridge, Cambridge, England; 4 Faculty of Medical Sciences, The University of the West Indies, Cave Hill, Barbados; 5 The University of the West Indies, Kingston, Jamaica; 6 The Sullivan Alliance, Alexandria, VA USA

**Keywords:** Disparity, Inequality, Social determinants, Prostate cancer, Systematic review, Caribbean

## Abstract

**Background:**

Prostate cancer remains the leading cause of cancer deaths among Caribbean men. However, little data exists on the influence of social factors on prostate cancer in the Caribbean setting. This article supports the 2011 Rio Political Declaration on addressing health inequalities by presenting a systematic review of evidence on the role of social determinants on prostate cancer in Caribbean men. It aims to determine the distribution, by known social determinants of health, of the frequency and adverse outcomes of prostate cancer among Caribbean populations.

**Methods:**

Observational studies reporting an association between a social determinant and prostate cancer frequency and outcomes were sought in MEDLINE, EMBASE, SciELO, CINAHL, CUMED, LILACS, and IBECS databases. Fourteen social determinants and 7 prostate cancer endpoints were chosen, providing 98 possible relationship groups exploring the role of social determinants on prostate cancer. Observational studies with > 50 participants conducted in Caribbean territories between 2004 and 2016 were eligible. The review was conducted according to STROBE and PRISMA guidelines. Random-effects meta-analyses were performed.

**Results:**

From 843 potentially relevant citations, 13 articles from 9 studies were included. From these included studies, 24 relationships were reported looking at 11 distinct relationship groups, leaving 90 relationship groups (92% of all relationship groups) unexplored. Study heterogeneity and risk of bias restricted results to a narrative synthesis in most instances. Meta-analyses showed more diagnosed prostate cancer among men with less formal education (*n* = 2 studies, OR 1.60, 95%CI 1.18–2.19) and among men who were married (*n* = 3 studies, OR 1.54, 95%CI 1.22–1.95).

**Conclusions:**

This review highlights limited evidence for a higher occurrence of diagnosed prostate cancer among Caribbean men with lower levels of education and among men who are married. The role of social determinants on prostate cancer among Caribbean men remains poorly understood. Improvements in study quantity and quality, and reduced variability in outcomes and reporting are needed. This report represents the current evidence, and provides a roadmap to future research priorities for a better understanding of Caribbean prostate cancer inequalities.

**Electronic supplementary material:**

The online version of this article (10.1186/s12889-018-5696-y) contains supplementary material, which is available to authorized users.

## Background

In 2015, prostate cancer accounted for about one-quarter of all male cancer deaths in the Caribbean, making it the leading cause of male cancer deaths and the third leading cause of male deaths overall [1]. The age-standardized mortality rate from prostate cancer among Caribbean men was estimated to be 50 per 100,000 in 2015, over twice the mortality seen in the USA and UK [1, 2]. Caribbean rates have increased by nearly 40% since 1990, in contrast to the decrease seen among many industrialised countries [1, 2]. Prostate cancer occurrence increases after age 40 and is more common among African-Americans and men with particular germline mutations [3, 4]. Evidence on the role of other factors - such as diet, hormone levels, obesity and social determinants - on prostate cancer onset and progression remains less conclusive [4].

Despite the overall high mortality from prostate cancer in the Caribbean, little is known about whether prostate cancer and its outcomes vary *within* Caribbean populations. The 2007 Port of Spain Declaration was affirmed by Caribbean Commonwealth Heads of Government to reduce the burden caused by noncommunicable diseases (NCDs) [5]. Describing Caribbean NCD variability and associated social drivers is relevant in guiding public health policy in reducing NCDs. This is underscored by the 2011 Rio Political Declaration through which countries have committed to monitor and address health inequities, and the World Health Organization (WHO) Commission on the Social Determinants of Health (CSDH) has emphasized the importance of research to accommodate these objectives [6, 7].

Research exploring social inequalities among men with prostate cancer in the UK and USA offers evidence for the influence of ethnicity, socioeconomic position (SEP) and occupational exposures [8–14]. However, the social determinants of prostate cancer among Caribbean populations have yet to be reviewed systematically. The aim of this review is therefore to determine the distribution, by known social determinants of health, of the incidence, prevalence, and adverse outcomes of prostate cancer among populations living in the Caribbean. This process is guided by the analytical framework used to examine the social determinants of specific conditions by the WHO CSDH [15].

## Methods

A study protocol (see Additional file 1) provides the full methodology details. The methods were guided by a previous systematic review of social determinants of diabetes [16] and an initial scoping review of prostate cancer.

### Eligibility criteria

Observational studies from 32 Caribbean territories were sought, which reported at least one relationship between a social determinant and prostate cancer frequency (incidence, prevalence) or prostate cancer outcome (cancer stage, grade, recurrence, survival, and mortality). Articles written in the four official Caribbean languages (English, Spanish, French, and Dutch) were included. Study samples of any age were included, and were sampled from the general population or from healthcare facilities. Studies including less than 50 men were excluded as unlikely to be fully representative of the general population. Guided by the PRISMA statement for transparent reporting of systematic reviews and meta-analyses with a focus on health equity, which recommends the “PROGRESS” checklist, the following social determinants were used: place of residence, race or ethnicity, occupation, gender, religion, education, socio-economic position (SEP), and social capital [17]. Reports published between January 2004 and December 2014 were originally sought for inclusion, with a recent review update to also include reports published in 2015 and 2016. This study has taken place within the context of a major review of regional and national policy responses in the Caribbean to chronic NCDs [18]; the review period was selected as relevant to the current situation and able to inform policy response.

### Search strategy, study selection, data abstraction

MEDLINE (via Pubmed), EMBASE (via Ovid), SciELO (via SciELO), CINAHL (via EBSCO), and CUMED, LILACS, and IBECS (via WHO Virtual Health Library) databases were searched [19–23] using Endnote as the reference management software [24]. The final search was conducted in July 2017. The search strategies used are detailed Additional file 2.

Studies were selected and data was abstracted independently by two reviewers (SH, CB). Titles and abstracts were screened to first identify articles that were potentially relevant. Then, full-texts of these potentially relevant articles were screened to identify articles for inclusion. An electronic data abstraction form was created (see Additional file 1) using the REDCap software [25], and its content guided by the STROBE statement on strengthening the reporting of observational studies in epidemiology and the PRISMA-Equity statement [26, 27]. Inconsistent screening and abstraction results were reviewed by an independent third party (NSG).

### Risk of bias assessment

STROBE and Cochrane guidelines (see Additional file 1) were jointly used to create a risk of bias was tool which was used to assess bias at the relationship level [26, 28]. Five domains were assessed:

- Confounding (ie: might a relationship be affected by an unmeasured confounder?)
- Participant selection (ie: is the sample representative of the target population?)
- Missing data (ie: is the data reasonably complete?)
- Outcome measurement (ie: is a social determinant/disease endpoint appropriately measured?)
- Selective reporting (ie: is a relationship selectively reported?).

Relationships and articles were classified as having serious, moderate, low, or unclear risk of bias. Two reviewers (CB, NSG) made an independent judgement on the overall risk of bias of each included relationship and article, with any discrepancies resolved through discussion.

### Synthesis of results

The review was planned as a narrative synthesis, with meta-analysis of quantitative evidence restricted to relationships reported by ≥ 2 studies classified as having low or moderate risk of bias. Key study details are presented, followed by a description of each association between a social determinant and either a measure of disease frequency or a measure of disease outcome (with each association being termed an ‘inequality relationship’). An evidence gap map (Fig. 2) was used to summarize the number and type of inequality relationships [29]. Random-effects meta-analyses were performed in recognition of the anticipated heterogeneity between studies. Relationships eligible for meta-analysis described cancer frequency and were summarised using odds ratios. Sensitivity analyses included studies classified as having high/unclear risk of bias. All quantitative summaries were performed using Stata statistical software (release 14, College Station, TX: StataCorp LP).

## Results

### Summary of included studies

**Fig. 1:**
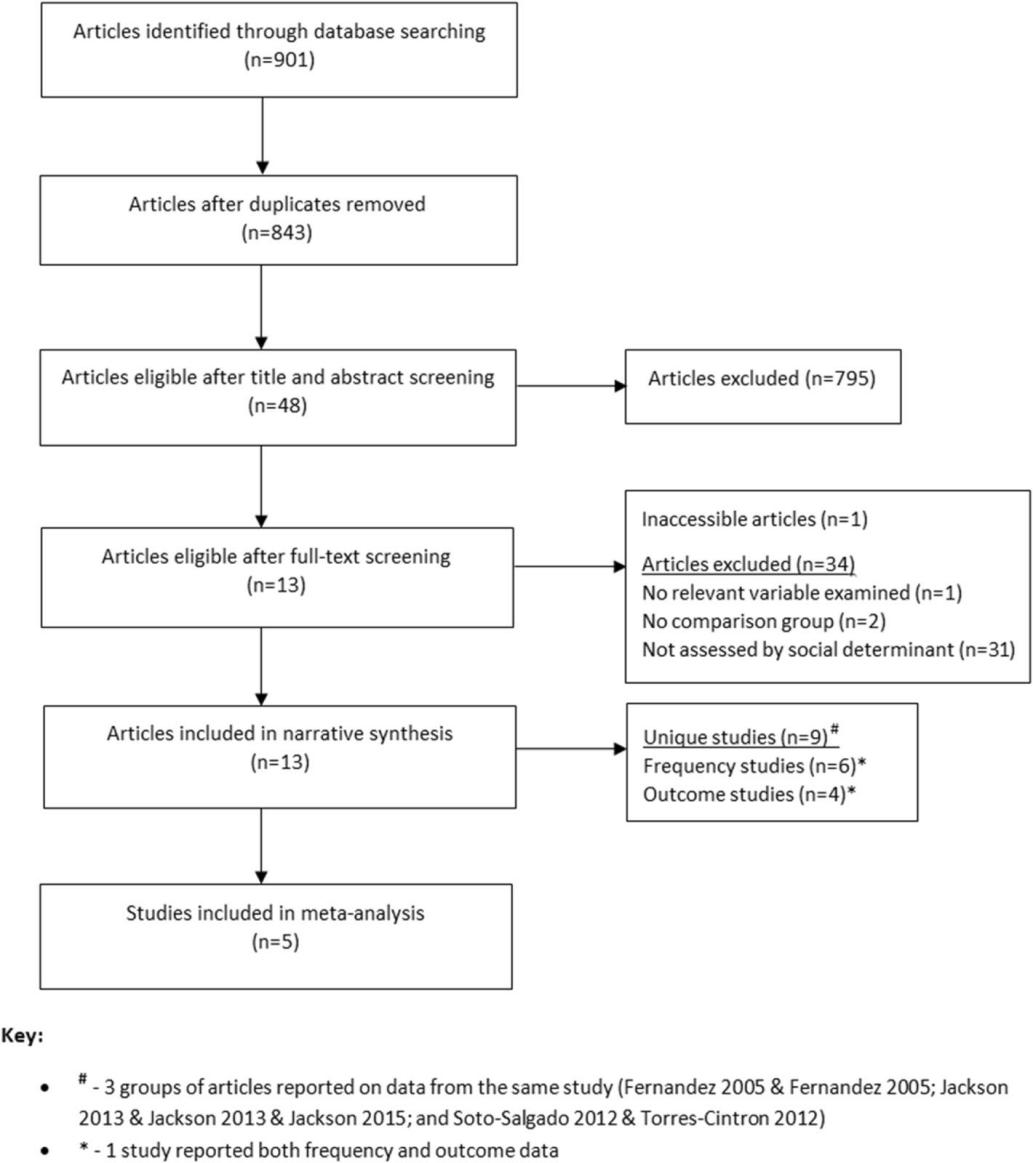
Flowchart of search strategy and article selection

Figure 1 presents a flowchart of articles identified, excluded, and included. From 843 identified articles, 13 articles reporting data from 9 unique studies were eligible for inclusion.

**Fig. 2:**
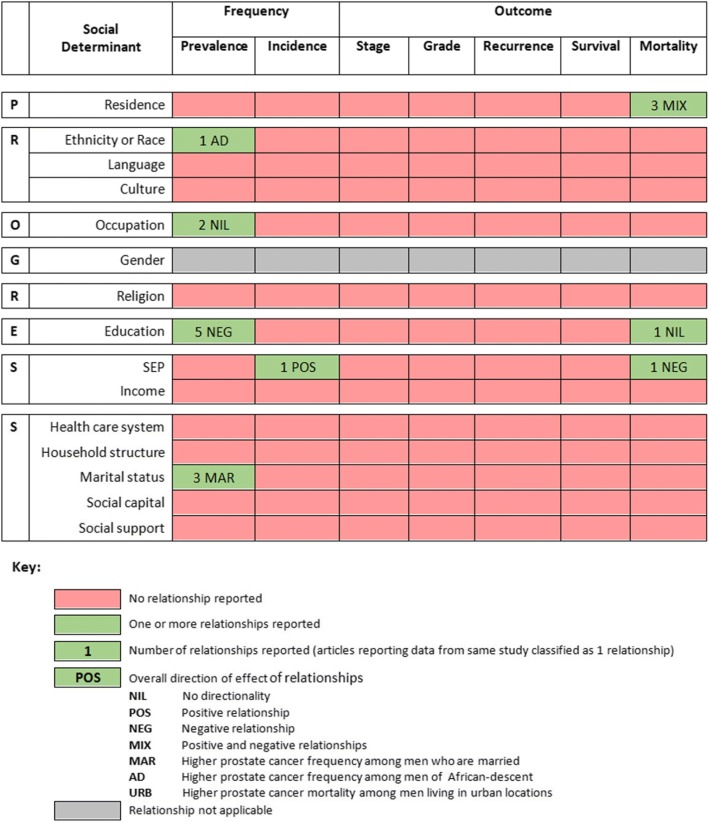
Summary of 17 unique relationships among 13 included articles [30–42]

Table 1 describes characteristics of the 13 included articles; all studies included 1 or more social determinant stratifications. Seven social determinants were examined by these articles. Of these 13 articles, 10 reported on prostate cancer frequency and 4 reported on prostate cancer outcomes. The studies were conducted in English-speaking (Barbados, Jamaica, Trinidad and Tobago); French-speaking (Guadeloupe) and Spanish-speaking (Cuba, Puerto Rico) Caribbean countries. Most articles reported on populations in Cuba, Jamaica, and Puerto Rico (*n* = 4 for each). Figure 2 summarizes the inequality relationships reported in the included articles.

**Table 1:** Characteristics of 13 included articles from the Caribbean describing the social distribution of prostate cancer frequency and outcomes [30–42]

Study-level characteristics							Inequality relationships reported		
Article (*n* = 13)	Study design	Sample size	Age	Study-base	Country	Measurement tool/source	Frequency	Outcome	Main findings
Bray, 2016 [42]	Registry-based	/	0–74	Population	Bahamas, Barbados, Cuba, Dominican Republic, Guadeloupe, Guyana, Haiti, Jamaica, Maritinique, Puerto Rico, Suriname, Trinidad & Tobago	/	/	Residence	Cumulative mortality risk per country is as follows - Puerto Rico (1.0%), Martinique (1.8%), Suriname (1.9%), Cuba (2.3%), Guadelope (2.7%), Bahamas (3.0%), Haiti (3.1%), Dominican Republic (3.3%), Jamaica (3.8%), Barbados (3.9%), Guyana (4.4%), Trinidad and Tobago (4.9%)
Fernández, 2005 [30, 31]^a^	Case-control	527	< 84	Health-facility	Cuba	histological/cytological test	Education	/	Proportions (cases, controls) are as follows - none (37.7, 32.3%), primary (38.8, 35.8%), technical (17.9, 24.4%), university (5.5, 7.5%). Regression results are as follows - none (ref), primary (OR 0.6, CI 0.4–1.1), technical (OR 1.1, CI 0.7–1.6), university (OR 0.7, CI 0.4–1.3).
							Ethnicity		Proportions (cases, controls) are as follows - white (50.9, 57.5%), black or mixed (49.1, 42.5%). Regression results are as follows - white (ref), black or mixed (OR 1.3, CI 0.9–1.9).
							Marital status		Proportions (cases, controls) are as follows - married (65.9, 63.8%), single (9.9, 15.0%), divorced (11.4, 11.8%), widower (12.8, 9.4%). Regression results are as follows - married (ref), single (OR 1.1, CI 0.6–1.9), divorced (OR 1.4, CI 0.7–2.9), widower (OR 0.7, CI 0.3–1.4).
							Occupation		Proportions (cases, controls) of Set 1 are as follows - worker (13.2, 25.7%), retired and not working (59.7, 64.2%), retired and working (26.7, 19.7%), unemployed (0.4, 0.4%). Regression results are as follows - workers (ref), retired and not working (OR 1.1, CI 0.7–1.8), retired and working (OR 1.6, CI 0.9–2.9), unemployed (OR 1.1, CI 0.1–18.4). Proportions (cases, controls) of Set 2 are as follows: qualified non-manual worker (15.0, 15%), qualified manual worker (31.5, 33.1%), administrative assistant (13.9, 11.8%), administrative worker (16.1, 16.1%), foreman (4.0, 6.7%), teacher (0.4, 0.8%), craftsman (2.2, 0.8%), shop keeper (6.6, 6.7%), professional (5.5, 7.5%), agricultural worker (4.8, 1.6%).
Fernández, 2005 [30, 31]^a^	Case-control	527	< 84	Health-facility	Cuba	histological/cytological test	Education	/	Proportions (cases, controls) are as follows - none (37.7, 32.3%), primary (38.8, 35.8%), technical (17.9, 24.4%), university (5.5, 7.5%).
							Ethnicity		Proportions (cases, controls) are as follows - white (52.2, 57.9%), black (24.1, 20.2%), mixed (23.7, 21.8%).
							Marital status		Proportions (cases, controls) are as follows - married (65.9, 63.8%), single (9.9, 15.0%), divorced (11.4, 11.8%), widower (12.8, 9.4%).
Jackson, 2012 [35]^b^	Case-control	435	40 to 80	Health-facility	Jamaica	histological test	Education	/	Proportions are as follows (all cases, high grade cases, low grade cases, controls) - primary or less (89.5, 83.8, 93.3, 79.9%), secondary (5.8, 8.0, 3.8, 14.6%), tertiary (4.7, 7.5, 2.9, 5.4%).
Jackson, 2013 [32]^b^	Case-control	402	40 to 80	Health-facility	Jamaica	histological test	Education	/	Proportions are as follows (all cases, high grade cases, low grade cases, controls) - primary or less (89.5, 83.8, 93.3, 79.9%), secondary (5.8, 8.0, 3.8, 14.6%), tertiary (4.7, 7.5, 2.9, 5.4%).
Jackson, 2015 [39]^b^	Case-control	472	41 to 80	Health-facility	Jamaica	histological test	Education	/	Proportions (cases, controls) are as follows - primary or less (90.3, 80.8%), secondary or higher (9.7, 19.2%). *p* = 0.003
McDonald, 2011 [36]^d^	Case-cohort	511	40 to 81	Region/community	Trinidad &Tobago	fine needle aspiration biopsy	Education	/	Proportions (cases, controls) are as follows - ≤11 years (80.2, 77.0%), > 11 years (19.8,% 23.0). *p* = 0.83.
							Marital status		Proportions (cases, controls) are as follows - ever married (85.4, 81.0%), never married (14.6, 19.0%). *p* = 0.48.
Multigner, 2010 [37]	Case-control	1294	adults	Population	Guadeloupe	histopathological test	Education	/	Proportions (cases, controls) are as follows - high school and higher (13.3, 10.7%), secondary (25.4, 31.9%), primary (61.4, 57.4%). *p* = 0.03.
Nemesure, 2013 [33]^d^	Case-control	1271	adults	Population	Barbados	histological test	Education	/	Means and standard deviations of total years of education are as follows - cases (11.9+/−3.9), controls (11.6+/−3.3). *p* = 0.21.
							Marital status		Proportions (cases, controls) are as follows - single or never married (15.4, 22.3%), married or living together (61.0, 52.0%), separated or divorced (10.0, 14.0%), widowed (8.6, 7.3%). *p* = 0.001.
							Occupation		Proportions (cases, controls) are as follows - professional/administration/management (25.9, 22.5%). *p* = 0.15.
Santana, 2011 [40]	Cross-sectional	NR	adults	Population	Cuba	Mortality Statistics Department of the Provincial Health Directorate Santiago de Cuba and the State Committee for Statistics (census)	/	Residence	The number of deaths and crude mortality rates (per 100,000) of prostate cancer are as follows - Contramaestre (21, 39.2), Mella (12, 66.2), San Luis (27, 59.3), II Frente (11, 52.9), Songo-La Maya (41, 85.8), Santiago (128, 53.0), Palma (31, 50.3), III Frente (4, 25.2), Guamá (10, 54.5)
Smit, 2007 [41]^d^	Prospective cohort	9824	35 to 79	Population	Puerto Rico	Puerto Rico cancer registry and Puerto Rico biostatistics registry	/	Education	Proportions for prostate cancer death cases and non-prostate cancer death cases are as follows - no formal schooling (9.6, 10.1%), grades 1–4 (30.5, 35.3%), grades 5–8 (34.7, 28.7%), attended/completed high school (13.2, 17.7%), more than high school (12.0, 8.2%). *p* = 0.09.
								Residence	Proportions for prostate cancer death cases and non-prostate cancer death cases are as follows - urban (28.1, 30.5%), rural (71.9, 69.5%). *p* = 0.52.
Soto-Salgado, 2012 [34]^c^	Registry-based	NR	45+	Population	Puerto Rico	Puerto Rico central cancer registry and surveillence, epidemiology, and end results (SEER) programme of the national cancer institute	SEP^I e^	SEP ^e^	Age-specific incidence per 100,000 is as follows - SEP1 (334.0), SEP2 (322.8), SEP3 (305.8), SEP4 (336.2), SEP5 (396.5). Ratio and CI of SEP5/SEP1 is 1.12, 1.04–1.21. Age-specific mortality per 100,000 is as follows - SEP1 (102.7), SEP2 (84.7), SEP3 (79.6), SEP4 (85.1), SEP5 (89.4). Ratio and CI of SEP5/SEP1 is 0.88, 0.07–1.02. Refer to article for age-specific rates.
Torres-Cintrón, 2012 [38]^c^	Registry-based	NR	45+	Population	Puerto Rico	Puerto Rico Central Cancer Registry (PRCCR) and Puerto Rico Department of Health	SEP^I e^	SEP ^e^	Age-specific incidence per 100,000 is as follows - SEP1 (334.0), SEP2 (322.8), SEP3 (305.8), SEP4 (336.2), SEP5 (396.5). Ratio and CI of SEP5/SEP1 is 1.12, 1.04–1.21. Age-specific mortality per 100,000 is as follows - SEP1 (102.7), SEP2 (84.7), SEP3 (79.6), SEP4 (85.1), SEP5 (89.4). Ratio and CI of SEP5/SEP1 is 0.88, 0.07–1.02. Refer to article for age-specific rates.

^a^These articles used data from the same Cuban study

^b^These articles used data from the same Jamaican study

^c^These articles used data from the same Puerto Rican study

^d^These articles are each components of larger studies: (Nemesure [33] - Prostate Cancer in a Black Population) [62], (Smit [41] - Puerto Rico Heart Health Program) [63], (McDonald [36] - Tobago Prostate Study) [64]

^e^Article authors defined SEP by 8 area-level socioeconomic indicators from the national census: unemployment rate, median annual household income, percentage of the population living below the poverty level, percentage of the population > aged 25 years with < 12 years of education, percentage of occupied housing units without a car, percentage of employed population aged > 16 years in white-collar occupations, percentage of occupied housing units without a telephone, and percentage of population fluent in both English and Spanish

/ - Not reported

I (in ‘Frequency’ column) - These studies examine prostate cancer frequency as incidence, rather than number of cases

There were 24 inequality relationships reported: 18 on prostate cancer frequency and 6 on prostate cancer outcomes. When articles reporting data from the same study are removed, the number of inequality relationships falls to 17: 12 on frequency and 5 on outcomes. There is a crucial evidence gap on the effects of social determinants on prostate cancer among Caribbean men. With 14 social determinants and 7 review outcomes, there were 98 unique inequality relationship groups that could have been reported. Just 8 (8%) of these relationship groups were reported by the 13 included articles, leaving 90 (92%) relationship groups without an evidence base.

### Risk of bias of included studies

**Fig. 3:**
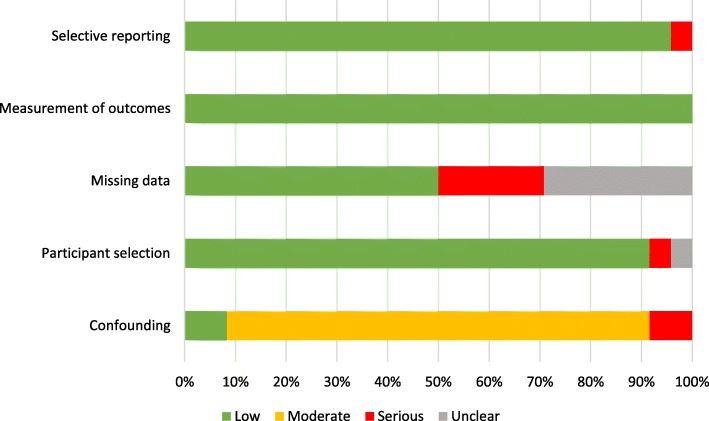
Proportion of risk of bias classifications of the 24 relationships across the 5 domains [30–42]

The risk of bias assigned to each of the 24 social determinant relationships is presented in Table 2. Of the 13 articles, 1 was classified as having low risk of bias, 9 as having moderate risk of bias, 1 as having serious risk of bias, and 1 as having unclear risk of bias. Figure 3 details the proportion of relationship classifications within each of the 5 risk of bias domains. Overall, lack of adjustment for confounding was the main contributor to an increased risk of bias, followed by non-disclosure or inadequate handling of missing data.

**Table 2:** Risk of bias assessments among 24 relationships from 13 included articles [30–42]

Article (*n* = 13)	Relationship (*n* = 24)		Bias domain					
	Endpoint	Social determinant	Confounding	Participant selection	Missing data	Measurement of outcomes	Selective reporting	Overall
Bray, 2016 [42]	Outcome	Residence	Moderate	Unclear	Unclear	Low	Low	Unclear
Fernández, 2005 [30, 31]^a^	Frequency	Education	Moderate	Low	Low	Low	Low	Moderate
	Frequency	Ethnicity	Moderate	Low	Low	Low	Low	Moderate
	Frequency	Marital Status	Moderate	Low	Low	Low	Low	Moderate
	Frequency	Occupation	Moderate	Low	Low	Low	Serious	Moderate
Fernández, 2005 [30, 31]^a^	Frequency	Education	Moderate	Low	Low	Low	Low	Moderate
	Frequency	Ethnicity	Moderate	Low	Low	Low	Low	Moderate
	Frequency	Marital Status	Moderate	Low	Low	Low	Low	Moderate
Jackson, 2012 [35]^b^	Frequency	Education	Moderate	Low	Unclear	Low	Low	Moderate
Jackson, 2013 [32]^b^	Frequency	Education	Moderate	Low	Serious	Low	Low	Moderate
Jackson, 2015 [39]^b^	Frequency	Education	Moderate	Low	Serious	Low	Low	Moderate
McDonald, 2011 [36]^d^	Frequency	Education	Low	Low	Low	Low	Low	Low
	Frequency	Marital Status	Low	Low	Low	Low	Low	Low
Multigner, 2010 [37]	Frequency	Education	Serious	Serious	Low	Low	Low	Serious
Nemesure, 2013 [33]^d^	Frequency	Education	Moderate	Low	Serious	Low	Low	Moderate
	Frequency	Marital Status	Moderate	Low	Serious	Low	Low	Moderate
	Frequency	Occupation	Moderate	Low	Serious	Low	Low	Moderate
Santana, 2011 [40]	Outcome	Residence	Serious	Low	Unclear	Low	Low	Serious
Smit, 2007 [41]^d^	Outcome	Education	Moderate	Low	Low	Low	Low	Moderate
	Outcome	Residence	Moderate	Low	Low	Low	Low	Moderate
Soto-Salgado, 2012 [34]^c^	Frequency	SEP	Moderate	Low	Unclear	Low	Low	Moderate
	Outcome	SEP	Moderate	Low	Unclear	Low	Low	Moderate
Torres-Cintrón, 2012 [38]^c^	Frequency	SEP	Moderate	Low	Unclear	Low	Low	Moderate
	Outcome	SEP	Moderate	Low	Unclear	Low	Low	Moderate

^a^These articles used data from the same Cuban study

^b^These articles used data from the same Jamaican study

^c^These articles used data from the same Puerto Rican study

^d^These articles are each components of larger studies: (Nemesure [33] - Prostate Cancer in a Black Population) [62], (Smit [41] - Puerto Rico Heart Health Program) [63], (McDonald [36] - Tobago Prostate Study) [64]

### Summary of included inequality relationships

#### Prostate cancer frequency

There were 18 inequality relationships examining the frequency of prostate cancer, reported by 10 articles across 5 social determinants: education (*n* = 8), ethnicity (*n* = 2), marital status (*n* = 4), occupation (*n* = 2), and SEP (*n* = 2) [30–39]. Prostate cancer frequency was defined as the number of cases by 8 articles and incidence rate by 2 articles [34, 38].

**Fig. 4:**
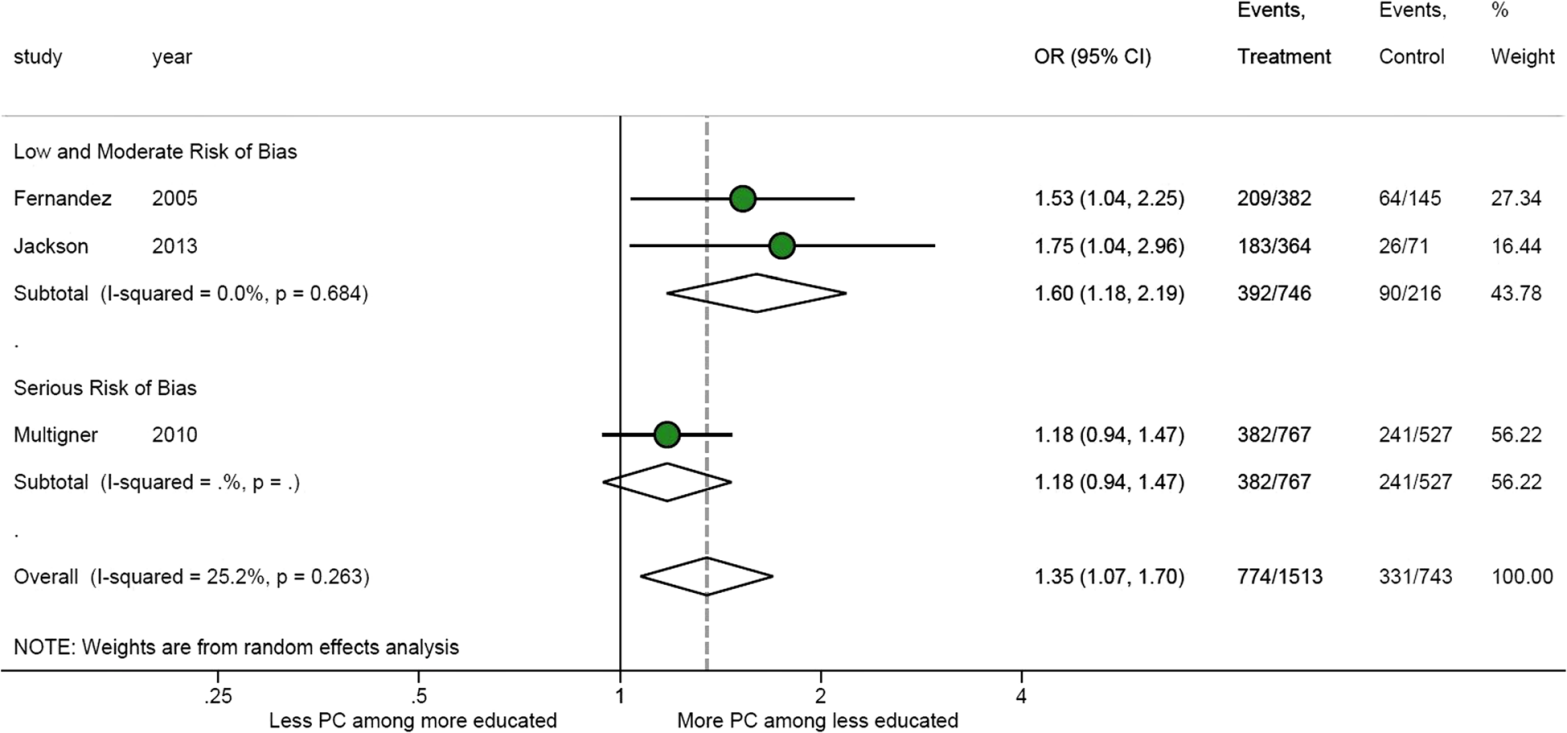
Meta-analysis of the relationship between incident cases of prostate cancer and education [31, 32, 37]

Six studies (8 articles) examined the association of prostate cancer frequency and education, 5 of which reported an increased frequency of prostate cancer among men with less formal education [30–33, 35–37, 39]. All studies used a case-control design, and relationships originated from Cuba (*n* = 2), Jamaica (*n* = 3), Barbados (*n* = 1), Guadeloupe (*n* = 1), and Trinidad and Tobago (*n* = 1). Seven relationships were of moderate risk of bias, while the single Guadeloupe relationship was of high risk of bias. Figure 4 presents a meta-analysis of the relationship between education and prostate cancer frequency; multiple articles reporting data from the same study were not included. Using studies classified as having low or moderate risk of bias [31, 32] and stratifying education as “primary or less” or “secondary or more”, results indicate that men with primary education or less were more likely to have had prostate cancer (OR 1.60, 95%CI 1.18–2.19). In a sensitivity analysis including 1 additional serious-risk study [37], the direction of effect remained but the pooled odds ratio reduced in size (OR 1.35, 95%CI 1.07–1.70).

**Fig. 5:**
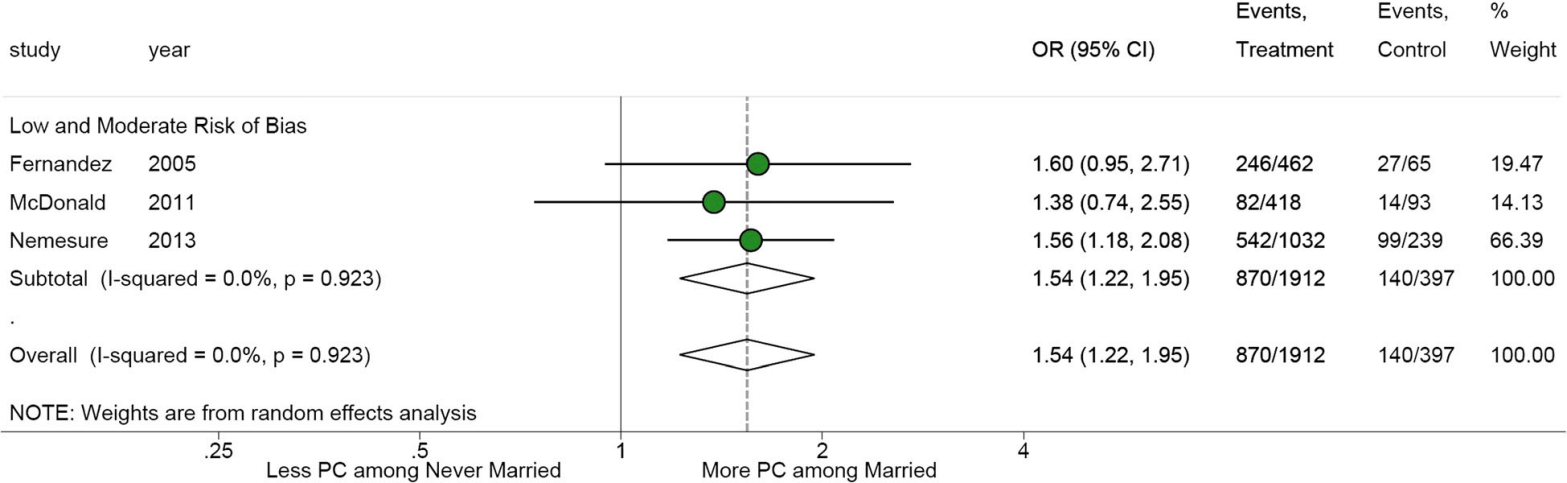
Meta-analysis of the relationship between incident cases of prostate cancer and marital status [31, 33, 36]

Four articles from 3 studies examined the age-adjusted association of prostate cancer frequency and marital status, showing higher prostate cancer frequency among married men [30, 31, 33, 36]. All studies used a case-control design and relationships originated in Barbados (*n* = 1), Cuba (*n* = 2), and Trinidad and Tobago (*n* = 1). Three relationships were classified as moderate risk of bias, while the Trinidad study was classified as having low risk of bias. Figure 5 presents the meta-analysis of the relationship between prostate cancer and marital status; multiple articles reporting data from the same study were not included. Stratifying marital status as “ever married” or “never married”, married men were more likely to have had prostate cancer (OR 1.54, 95%CI 1.22–1.95) [31, 33, 36].

Two articles from 1 Cuban case-control study examined the age-adjusted association of prostate cancer frequency and ethnicity, reporting that Black Cuban men were more likely to have prostate cancer than White Cuban men (OR 1.3, CI 0.9–1.9; *p* = 0.05) [30, 31]. These relationships were classified as having moderate risk of bias. Two articles from Cuba and Barbados examined the relationship between prostate cancer frequency and occupation, neither reporting an association [30, 33]. Both relationships were of moderate risk of bias. Two articles from 1 Puerto Rican study examined the association between prostate cancer frequency and SEP [34, 38]. SEP was defined by 8 residential area level indicators and both articles reported higher rates of prostate cancer among men with higher SEP (SEP5 (highest)/SEP1 (lowest) ratio 1.12, 95% CI 1.04–1.21).

#### Prostate cancer outcomes

There were 6 inequality relationships reporting on prostate cancer outcomes, reported by 5 articles across 3 social determinants: education (*n* = 1), residence (*n* = 3), and SEP (*n* = 2) [34, 38, 40–42]. Mortality was the only outcome reported and studies were cohort, cross-sectional and registry-based designs. Studies originated from Puerto Rico (*n* = 4) and Cuba (*n* = 1), with the remaining study examining 12 Caribbean territories collectively. All relationships were classified as moderate risk of bias, except for the single Cuban study examining residence which was classified as serious risk of bias and the Caribbean-wide study examining residence which was classified as unclear risk of bias.

The single study examining education and mortality (Puerto Rico) reported no association [41]. Three studies examining mortality and area of residence offered limited information [40–42]. The Cuban study did not differenciate rural/urban divides, while the Puerto Rican study reported no difference in mortality in urban versus rural locations. The Caribbean-wide study did not formally assess mortality differences in urban versus rural settings, but did list mortality rates by country. Barbados (3.9%), Guyana (4.4%), and Trinidad and Tobago (4.9%) were reported to have the highest cumulative mortality risks, while Puerto Rico had the lowest risk of 1.0% [42]. Two articles from 1 Cuban study examined the association between mortality and SEP, reporting that men with lower SEPs had higher age-adjusted prostate cancer mortality (SEP5 (highest)/SEP1 (lowest) ratio 0.88, 95% CI 0.07–1.02) [34, 38].

## Discussion

### Summary of evidence

This systematic review has examined the extent of evidence on the influence of social determinants of health on prostate cancer frequency and adverse outcomes in the Caribbean. Thirteen articles from 9 separate studies were included. With 14 possible social determinants and 7 chosen prostate cancer endpoints, there were 98 possible ways (relationship groups) of exploring the role of social determinants on prostate cancer. From the included studies, 24 relationships were reported looking at 8 distinct relationship groups, leaving 90 relationship groups (92% of all groups) without an evidence base.

Most articles were classified as having moderate risk of bias, mostly because of failures to adjust for important potential confounders, which limited interpretation. A key consideration for social determinant studies is the recognition of interrelationships among the social determinants themselves. For instance, ethnicity often contributes to our understanding of inequalities between population subgroups. Using an international example, African Americans are often disadvantaged compared to Caucasian Americans in terms of education, occupation, and income; with each of these social determinants associated strongly with access to healthcare and later health effects [43].

In this review, prostate cancer occurrence was consistently higher in men with lower levels of education, which conflicts with some international evidence [44–46]. In a US study, for example, higher education was associated in all ethnic groups with a higher prostate cancer incidence, attributed partly to greater use of health and screening services [45]. Inequalities in screening uptake can be influenced by differences in health-seeking behaviour or access to healthcare provision [13, 34, 44]. For instance, increased screening by more affluent social groups is reflected in our finding that Caribbean men in higher SEPs had a higher incidence of prostate cancer [34, 38]. A more recent Caribbean study supports this notion of SEP inequalities – this time through income inequalities – with screening for prostate cancer reported to be higher among Dominican Republicans with health insurance coverage [47]. Our conflicting finding for education may reflect the dynamics of different healthcare systems between Puerto Rico (SEP study) and countries examining education (Cuba, Jamaica, Barbados, Guadeloupe, Trinidad and Tobago); the interplay of proxies defining SEP; or perhaps a failure of the included studies to fully explore interrelationships between competing social determinants (such as education and ethnicity, or education and social support).

Similar to other settings [48, 49], marriage was associated with a higher reported occurrence of prostate cancer in Caribbean men [30, 31, 33, 36]. The social support extended by marriage is thought to promote health-seeking behaviour, leading to a greater chance of diagnosis [50, 51]. Without this social support, health-seeking reluctance leads to delayed diagnosis and a higher risk of adverse outcomes. On the other hand, a growing body of evidence explains that men with fewer sexual partners and subsequent lower rates of venereal disease, as well as men with a higher ejaculation frequency – both likely conditions of a typical married life - could lower the risk of developing prostate cancer [32, 52–58]. The social consequences of marriage might therefore also include a lower prostate cancer mortality [56].

Our results highlighting an increased prostate cancer frequency among men of African descent are supported by a large body of evidence emphasizing the importance of ethnicity as a social determinant of lifestyle risk factors and health status [12, 34, 43, 45, 57, 58]. However, a biological component may also be at play. Genotypes associated with prostate cancer incidence and prognosis, such as Steroid 5 alpha-reductase and Cytochrome P450 3A4, are more commonly seen in persons of African origin [34, 43, 58, 59]. The predominance of an ‘African genome’ in many Caribbean populations could play a role in the regional burden of prostate cancer, and its interplay with social determinants remains an important area for further research [34, 58, 59].

Evidence for associations between social determinants and prostate cancer outcomes is sparse. Mortality from prostate cancer was reported to be higher among men with a lower SEP [34, 38]. This may be related to reduced availability of or access to screening and other health care services, as well as reduced health literacy impacting on cancer stage at diagnosis and treatment adherence [43–45]. Notably, the Cuban study examining SEP calculated SEP using community-level measurements (see Table 1 footnote); other Caribbean settings, using individual-level SEP, may have different findings.

An important consideration when examining differences between studies from different Caribbean countries is country-level healthcare governance. Two locations of our included studies - Guadeloupe and Puerto Rico - are territories of France and the USA respectively, with the commensurate possibility of greater healthcare resources. For instance, in a recent examination of life expectancy in the Caribbean, Martinique and Guadeloupe (territories of France) had the highest Caribbean life expectancies and the largest improvements in life expectancy over 40 years [60]. However, as it applies to the included studies of this review from non-sovereign Caribbean countries [34, 37, 38, 41, 42, 61], they either showed no difference in directionality from relationships of independent Caribbean territories or their relationships were not reported by other independent Caribbean territories to allow comparison.

### Limitations

This review is limited by a small number of articles eligible for inclusion (*n* = 13), particularly for prostate cancer outcomes. The Caribbean – considered as one region geographically – indeed has country-level variation in social determinants that are possibly masked by this grouping and analysis. Publication bias is an important concern as limited resources restricted grey literature searching. At the study-level, validity of results is limited by the moderate or serious risk of bias assigned to many of the included studies. Country-level information on screening and access to treatment, such as prostate-specific antigen screening rates and wait times for diagnosis or treatment, are important potential confounders that were not assessed in the individual studies.

## Conclusion

This review suggests a higher occurrence of prostate cancer among Caribbean men with lower levels of education (OR 1.60, 95%CI 1.18–2.19) and among married men (OR 1.54, 95%CI 1.22–1.95). Statements on the role of other social determinants in the Caribbean must be tempered by a paucity and limited quality of evidence. The WHO CSDH has highlighted the role of health research in understanding health inequities, and Caribbean countries have committed to addressing these inequities [6, 7]. Although the need for more research in this area is acknowledged, this effort to improve the evidence base should include an attempt at standardizing reporting guidelines for observational studies of inequality. For systematic reviews of observational evidence, the development of a validated risk of bias assessment tools is an imperative.

## Additional files

Additional file 1: Study Protocol. (DOCX 4024 kb)

Additional file 2: Search Strategies. (DOCX 16 kb)

## Abbreviations

CINAHL: Cumulative Index of Nursing and Allied Health Literature
CSDH: Commission on the Social Determinants of Health
CUMED: Cuba Medicina
EMBASE: Excerpta Medica Database
IBECS: Índice Bibliográfico Español en Ciencias de la Salud
LILACS: Latin American and Caribbean Health Sciences
MEDLINE: Medical Literature Analysis and Retrieval System Online, or MEDLARS Online
NCD: Noncommunicable disease
SciELO: Scientific Electronic Library Online
SEP: Socioeconomic Position
STROBE: Strengthening the Reporting of Observational studies in Epidemiology
USCAHDR: United States Caribbean Alliance for Health Disparities Research Group
WHO: World Health Organization
