# Supplementary material for: Social determinants of prostate cancer in the Caribbean: a systematic review and meta-analysis
Source: BMC Public Health. 2018 Jul 20;18:900. doi: 10.1186/s12889-018-5696-y (PMC6053791; doi:10.1186/s12889-018-5696-y)
Supplement: Supplementary file 1 — Study Protocol. (DOCX 4024 kb) [file 12889_2018_5696_MOESM1_ESM.docx]

# Study Protocol

# Title

Social determinants of breast and prostate cancers in populations living in the Caribbean: a systematic review and meta-analysis.

# Background

Each year, the World Health Organization and International Agency for Research on Cancer supports the Union for International Cancer Control to promote ways to ease the global burden of cancer.(1) Approximately 30% of cancers can be prevented when taking into consideration risk factors such as diet or physical inactivity, which are themselves influenced by social determinants of health.(2,3) To date, there has been no published systematic review of research conducted in the Caribbean that examines the social determinants of various types of cancers. Breast and prostate cancers are the leading causes of death from cancers in the Caribbean, and for this reason, a systematic review will be done on each cancers.(4,5)

Among females in the Caribbean, breast cancer was the eighth leading cause of death, accounting for the greatest proportion deaths and disability adjusted life years (DALYs) among all cancers in 2013.(4,5) Additionally, breast cancer attributed for 1.4 million DALYs in 2012.(6) Age-standardized mortality rates in the Caribbean have increased by 37% to 20.6 per 100,000 since 1990, in contrast to the decrease seen among industrialised countries.(4,5) There have been shown to be differences across socioeconomic status in cancer incidence and mortality.(7,8)

In 2010, prostate cancer accounted for about one-quarter of all male cancer deaths in the Caribbean, making it the leading cause of male cancer deaths and the fifth leading cause of male deaths overall. (4,5) Of the top ten causes of males mortality in the Caribbean in 1990, prostate cancer was estimated to have the highest median percent increase to 2010.(4,5) Age-standardized mortality rates from prostate cancer among Caribbean males were estimated to be 39 per 100,000 in 2010, around twice the mortality seen in the USA and UK.(4,5) Age-standardised mortality rates in the Caribbean have increased since 1990, in contrast to the decrease seen among industrialised countries.(4,5)

In spite of this burden, it is still not clear how social determinants of health may affect prostate and breast cancer risk factors, occurrence, and outcomes in the Caribbean. Exploring the social determinants of health that may be associated with breast and prostate cancers will aid in responding strategically to the increasing burden of these cancers in the Caribbean.

# Systematic Review Framework

The 2011 World Conference on Social Determinants of Health and its subsequent Rio Political Declaration on Social Determinants of Health rightfully recognize the critical role that social determinants play in the distribution of noncommunicable diseases such as cancer, as well as government commitment to improving sustainable development and health equity using the social determinants approach.(2) The associated challenge of identifying and addressing health inequities between populations and groups, such that the World Health Organization’s Global Action Plan on Prevention and Control of Noncommunicable Diseases 2013-2020 can be utilized.(2,9) Despite the heavy burden of cancer on regional morbidity rates, there is no published systematic review of research conducted in the Caribbean that examines the social determinants of different types of cancers. Such a review can inform regional preventive strategies for cancers and their complications, and also identify areas for further research.

The planning of this systematic review was guided by the analytical framework used to examine the social determinants of specific conditions by the WHO Commission on the Social Determinants of Health.(2) The framework has five levels and three dimensions, as shown in Figure 1 below. The Commission’s starting point to using this framework was to examine differential health outcomes by markers of social and economic status (such as gender, ethnicity, education, and occupation), and then to look upstream to investigate where these differences originate. After analyzing the determinants in this way, contributors to the WHO Commission then examined potential interventions to address the determinants, and suggested indicators to be measured in order to assess the success of those interventions (the ‘intervene’ and ‘measure’ dimensions in Figure 1).

Figure 1: Analytical Framework for Priority Public Health Conditions used by the WHO Commission on Social Determinants. Taken from: Blas E, Kurup AS, editors. Equity, social determinants, and public health programmes [Internet]. World Health Organization: World Press; 2010(2)


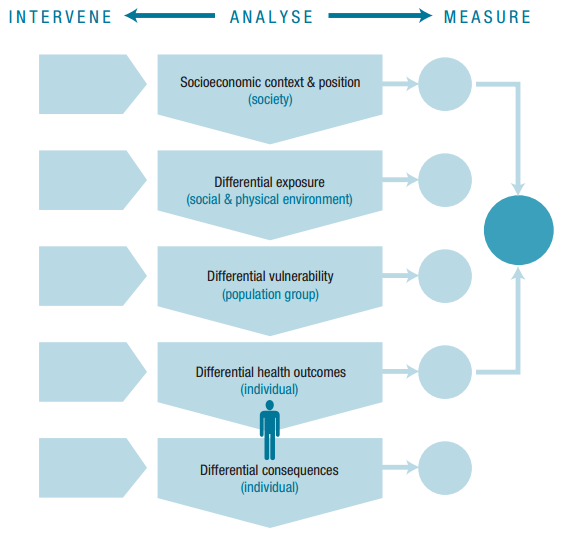


Reviewing the literature across the five levels and three dimensions is beyond the scope of a single review. Thus, our aim of this review is to provide a solid foundation for further work on health inequities of cancer in the Caribbean by reviewing the social distribution of the prevalence and incidence, risk factors (‘vulnerabilities’), and complications (‘consequences’) of cancer.

There is a clear rationale underpinning the chosen inclusion criteria for this review. The top male and female cancer within the Caribbean, according to the Global Burden of Disease study mortality rates, were selected for this review.(4) All ages are selected for the population to keep the review as broad as possible. A sample size limit of >50 participants or respondents is used as it is expected that studies with a small sample size will be less likely to be representative of the population. The study types included were all observational studies as the review is aimed at assessing the distribution, risk factors, and adverse outcomes of cancers, rather than interventions. The risk factors, frequency measures, and outcome measures to be assessed are listed in Table 1. They were selected specifically to ensure that the items were broadly scoped to capture as many studies as possible. Eligible risk factor were identified using three compendiums of evidence-based information: The Global Burden of Disease Consortium, UpToDate, and Cancer Epidemiology and Control.(10–12) The social determinants selected for the inclusion criteria are also listed in Table 1, and were guided by an extension of the PRISMA statement for reporting systematic reviews with a focus on health equity. (13)

# Review Question

**Primary Question:** What is the distribution, by known social determinants of health, of the risk factors, frequency, and adverse outcomes of breast cancer and prostate cancer in populations living in the Caribbean?

**Secondary Question:** What are the implications of these distributions for reducing and preventing further health inequities within the Caribbean region?

# Methods

## Inclusion & Exclusion Criteria

**Inclusion Criteria:**

- Participants/respondents resident in the Caribbean region, inclusive of the following countries: Anguilla, Antigua and Barbuda, Aruba/Bonaire/Curacao, The Bahamas, Barbados, St. Bart’s, Belize, Cayman Islands, Cuba, Dominica, Dominican Republic, St. Eustatius, French Guiana, Grenada, Guadeloupe, Guyana, Haiti, Jamaica, St. Kitts and Nevis, St. Lucia, St. Martin, St. Maarten, Martinique, Montserrat, Puerto Rico, St. Vincent and The Grenadines, Saba, Suriname, Trinidad & Tobago, Turks & Caicos, and the Virgin Islands (US and British)
- Observational studies, both published and unpublished if captured in the electronic search.
- Studies which define cancer as diagnosed through any cancer screening tool.
- Sample size >50.
- Age of study participants: all.
- Studies describing the distribution of >1 factors in rows (A), (B) or (C) in the Table 1 below by >1 social factors in row (D).

Table 1: Key variables to be abstracted and collected.

| **Group** | **Factor Categories** | **Factors Being Examined** |
| --- | --- | --- |
| **A** | Disease measurements | incidence, prevalence |
| **B** | Risk factors | **Breast cancer:** alcohol intake; high dietary sugar; overweight/obesity; physical inactivity; therapeutic ionizing radiation; late age at first pregnancy; low parity; low/no breastfeeding  **Prostate cancer:** high dietary calcium |
| **C** | Outcomes | grade, stage, recurrence, survival, mortality (cancer-specific, all-cause) |
| **D** | Social distribution | age*, race/ethnicity, language, education, occupation, income/wealth, culture, religion, social capital, social support, residence, infrastructure, healthcare systems |

* - ‘Age’ is not applied to BMI as a risk factor, disease measurements or outcomes as it is biologically inherent to these variables.

**Exclusion Criteria:**

- Intervention studies, narrative review studies, commentaries, case series, qualitative studies and single case reports.
- Studies in which describe the relationships of interest within sub-populations that are not representative of the general population (eg: patients in renal failure).
- Literature on Caribbean diaspora (as opposed to populations living within the Caribbean).
- Literature that is written in any other language than English, Dutch, Spanish and French.
- Non-human studies.
- Sample size <50.

## Search Strategy

The search strategies for breast cancer and prostate cancer according to the specifications of the Pubmed search engine are detailed in Appendix A; these will be adapted as necessary to the syntax of other search engines.

- MEDLINE (via Pubmed): National Library of Medicine’s journal citation database of biomedical and life sciences journal articles.(14)
- EMBASE (via Ovid): Elsevier’s international database with in depth coverage of pharmacology, pharmaceutical science, clinical research, basic biomedical science, veterinary science and extensive allied health topics.(15)
- SciELO: Electronic library covering a selected collection of Brazilian scientific journals, being developed by FAPESP - Fundação de Amparo à Pesquisa do Estado de São Paulo, in partnership with BIREME - the Latin American and Caribbean Center on Health Sciences Information.(16)
- CINAHL (via EBSCO): EBSCO’s database indexing of the top nursing and allied health literature, covering nursing, biomedicine, health sciences librarianship, alternative/complementary medicine, consumer health and seventeen allied health disciplines.(17)
- CUMED (via WHO Virtual Health Library): Bibliographic database developed by the National Medical Library and cooperating institutions of the national network of health information with records from Cuban medical and allied sciences published in Cuba or abroad.(18)
- LILACS (via WHO Virtual Health Library): Database of the Latin American and Caribbean of Health Sciences Information System.(18)
- IBECS (via WHO Virtual Health Library): Biographic Index on Health Sciences from Spain, a potential source of Spanish language publications from the Caribbean.(18)

The publication dates for the full review span a 10 year period - from January 1st, 2004 through December 31st, 2014. This period was chosen as it sandwiches the 2007 Port of Spain Declaration, and studies published more than 10 year ago were considered too old to inform current policy on social determinants. The search terms for the social determinants are guided by the extension of the PRISMA statement for reporting systematic reviews with a focus on health equity.(19) The statement recommends using the PROGRESS-Plus checklist, which includes place of residence, race/ethnicity/culture/language, occupation, gender/sex, religion, education, socio-economic status, social capital and any other possible factors. All other search terms were conceptualized through thorough broad research on cancer studies to identify key indicators. Age was examined for risk factors only, due to its biological association with cancer frequency and outcomes.

## Study Selection

All studies selected for the systematic review will be downloaded into Endnote reference manager.(20) Study selection will be conducted in two steps by two reviewers:

1. Initial screening of titles and abstracts against the inclusion criteria to identify potentially relevant studies.
2. Secondary screening of the full-text studies identified as potentially relevant in the initial screening.

All studies will be reviewed by two reviewers. In instances where Step 1 is impossible to complete with only the title and abstract, the full-text is to be retrieved and screened as stated in Step 2. In instances where there is still poor clarity on whether to include a study, the study will be forwarded to an independent third party for consensus. The numbers of articles reviewed, selected, and excluded at each stage will be documented according to the flowchart depicted below in Figure 2.

Figure 2: Literature screening process according to the 2009 PRISMA flowchart template.

Studies included in qualitative synthesis
(n = )

Full-text articles excluded, with reasons
(n = )

Full-text articles assessed for eligibility
(n = )

Additional records identified through other sources
(n = )

Records identified through database searching
(n = )

## Identification

## Screening

## Eligibility

## Included

Studies included in quantitative synthesis (meta-analysis)
(n = )

Records excluded
(n = )

Records screened
(n = )

Records after duplicates removed
(n = )

## Data Extraction

Studies that pass both steps of the study selection process will be eligible for data extraction. Each full-text study will be independently data-extracted by two reviewers. Any discordance in data extraction will also be resolved by a third party reviewer. A data extraction form has been created (see Appedix B) in RedCap software in order to manage the data.(21) This form is designed to extract key study characteristics and findings relevant to the primary research question. It has also been designed to enable an assessment of risk of bias inherent in each study (See appendix C for details on our risk of bias assessment). The content of the data abstraction form has been guided by the STROBE statement on reporting observational epidemiology and by the PRISMA statement on systematic reviews concerning health equity.(13,22)

Broadly, data items extracted from the included articles fall into one of the following information groups: basic study details (article title, author, publication year, study design, country/countries of data collection etc); risk factor details; disease details; adverse outcome details. The social determinants examined, tools/units of measurement, statistical techniques employed, results, confounders controlled, and assessment of risk of bias were depicted for each group. Should a study not have sufficient information required to fill out the data abstraction form to completion, that study will still be included in the review, but categorized as such.

Quality Assessment
Risk of bias will be assessed according a tool adapted from STROBE and Cochrane guidelines (see Appendix C).(22,23) Bias is to be assessed in 5 domains at the relationship level: confounding (might a relationship be affected by an unmeasured confounder?), participant selection (is the sample representative of the target population?), missing data (is the data reasonably complete?), outcome measurement (is a social determinant/disease endpoint appropriately measured?), selective reporting (is a relationship selectively reported?). Studies will be classified as having serious, moderate, low, or unclear risk of bias. Two reviewers will make an independent judgement on the overall risk of bias of each included article, considering each domain as equally important and the likely direction and magnitude of the bias from each domain. Discrepancies will be discussed by the two reviewers to achieve consensus.

Data Analysis
The review is planned as a narrative synthesis of evidence, with meta-analysis of quantitative evidence restricted to studies classified as having a low and moderate risk of bias. Sensitivity analyses will be conducted with any relevant high-risk articles.

For the narrative synthesis, key study-level information will be summarized for all studies. Variable-level information will be summarized, focusing separately on associations between social determinants and risk factors, between social determinants and cancer frequency, then between social determinants and cancer outcomes. To summarize quantitative information, a random-effects meta-analysis will be used to recognize for the anticipated heterogeneity between studies. The I-squared value will be reported to quantify heterogeneity.(23) Rate ratios will be used to report social determinant differences in cancer incidence or mortality. Odds ratios will be used to report social determinant differences in cancer prevalence. Hazard ratios will be used to report social determinant differences in cancer recurrence and survival. Cancer grade and stage will be converted to dichotomous data and reported using odds ratios.

# Plans for Dissemination

It is expected that the findings from the scoping review will be submitted for peer-reviewed publication. In addition, findings will be shared at Caribbean regional meetings such as the Caribbean Health Research Council’s annual meeting, as well as any relevant international Conferences.

# References

1. World Health Organization. Cancer [Internet]. WHO. [cited 2014 Oct 28]. Available from: http://www.who.int/cancer/en/

2. Blas E, Kurup AS, editors. Equity, social determinants, and public health programmes [Internet]. World Health Organization: World Press; 2010 [cited 2014 Oct 27]. 291 p. Available from: http://whqlibdoc.who.int/publications/2010/9789241563970_eng.pdf

3. World Health Organization. Cancer prevention [Internet]. WHO. 2015 [cited 2014 Oct 28]. Available from: http://www.who.int/cancer/prevention/en/

4. Lozano R, Naghavi M, Foreman K, Lim S, Shibuya K, Aboyans V, et al. Global and regional mortality from 235 causes of death for 20 age groups in 1990 and 2010: a systematic analysis for the Global Burden of Disease Study 2010. The Lancet. 2012 Dec;380(9859):2095–128.

5. Institute for Health Metrics and Evaluation. GBD Compare [Internet]. Institute for Health Metrics and Evaluation. 2013 [cited 2016 Mar 13]. Available from: http://vizhub.healthdata.org/gbd-compare/arrow

6. World Health Organization. Global Health Observatory data repository [Internet]. World Health Organization. 2015 [cited 2015 Jan 8]. Available from: http://apps.who.int/gho/data/view.main.DALYNUMWBDCPLACV?lang=en

7. Hiatt RA, Pasick RJ, Stewart S, Bloom J, Davis P, Gardiner P, et al. Cancer screening for underserved women: the Breast and Cervical Cancer Intervention Study. Cancer Epidemiol Biomark Prev Publ Am Assoc Cancer Res Cosponsored Am Soc Prev Oncol. 2008 Aug;17(8):1945–9.

8. Hiatt RA, Breen N. The social determinants of cancer: a challenge for transdisciplinary science. Am J Prev Med. 2008 Aug;35(2 Suppl):S141-150.

9. World Health Organization. Global action plan for the prevention and control of noncommunicable diseases 2013-2020 [Internet]. WHO Press; 2013 [cited 2014 Jun 2]. Available from: http://apps.who.int/iris/bitstream/10665/94384/1/9789241506236_eng.pdf

10. Lim SS, Vos T, Flaxman AD, Danaei G, Shibuya K, Adair-Rohani H, et al. A comparative risk assessment of burden of disease and injury attributable to 67 risk factors and risk factor clusters in 21 regions, 1990–2010: a systematic analysis for the Global Burden of Disease Study 2010. The Lancet. 2012 Dec;380(9859):2224–60.

11. Sartor A. Risk factors for prostate cancer [Internet]. UpToDate. 2014 [cited 2015 Jul 17]. Available from: http://www.uptodate.com/contents/risk-factors-for-prostate-cancer?source=search_result&search=prostate+cancer+risk+factors&selectedTitle=1~150#H11

12. Schottenfeld D, Jr JFF, editors. Cancer Epidemiology and Prevention. 3rd ed. Oxford University Press; 2006. 1416 p.

13. Welch V, Petticrew M, Tugwell P, Moher D, O’Neill J, Waters E, et al. PRISMA-Equity 2012 extension: reporting guidelines for systematic reviews with a focus on health equity. PLoS Med. 2012;9(10):e1001333.

14. U.S. National Library of Medicine. Fact Sheet MEDLINE, PubMed, and PMC (PubMed Central): How are they different? [Internet]. 2015 [cited 2015 Aug 13]. Available from: http://www.nlm.nih.gov/pubs/factsheets/dif_med_pub.html

15. Elsevier R&D Solutions. Embase Fact Sheet [Internet]. Elsevier B.V.; 2015 [cited 2015 Aug 13]. Available from: http://www.elsevier.com/__data/assets/pdf_file/0016/59011/R_D-Solutions_Embase_Fact-Sheet_DIGITAL.pdf

16. SciELO, FAPESP, BIRME. SciELO [Internet]. SciELO. n.d. [cited 2015 Aug 13]. Available from: http://www.scielo.br/

17. EBSCO. CINAHL Database [Internet]. 2015 [cited 2015 Aug 13]. Available from: https://health.ebsco.com/products/the-cinahl-database

18. Centro Nacional de Información de Ciencias Médicas, Infomed. Bibliographic Databases [Internet]. BVS Cuba: Biblioteca Virtual en Salud. n.d. [cited 2015 Aug 13]. Available from: http://www.bvsite.sld.cu/php/level.php?lang=en&component=30&item=3

19. Centre for Reviews and Dissemination. Systematic reviews: CRD’s guidance for undertaking reviews in health care [Internet]. Centre for Reviews and Dissemination; 2008 [cited 2014 Oct 29]. Available from: https://www.york.ac.uk/media/crd/Systematic_Reviews.pdf

20. EndNote [Internet]. Philadephia: Thomson Reuters; 2014 [cited 2015 Nov 10]. Available from: www.endnote.com

21. Harris PA, Taylor R, Thielke R, Payne J, Gonzalez N, Conde J. Research electronic data capture (REDCap) - A metadata-driven methodology and workflow process for providing translational research informatics support. J Biomed Inform. 2009;42(2):377–81.

22. von Elm E, Altman DG, Egger M, Pocock SJ, Gøtzsche PC, Vandenbroucke JP, et al. The Strengthening the Reporting of Observational Studies in Epidemiology (STROBE) statement: guidelines for reporting observational studies. J Clin Epidemiol. 2008 Apr;61(4):344–9.

23. A Cochrane risk of bias assessment tool: for non-randomized studies of interventions (ACROBAT-NRSI), version 1.0.0 [Internet]. The Cochrane Collaboration; 2014 Sep [cited 2015 Nov 11]. Available from: http://www.riskofbias.info

# Appendices

## Appendix A – Search Strategies for Pubmed Search Engine

Notes: Words that could be author names were restricted to non-author fields. Truncation (*) was not used in cases where the non-truncated word created a broader search because it triggers a MeSH term and automatically includes the pluralized form. Otherwise, both the truncated and non-truncated MeSH terms were used. Limits used: Human-only, date range = January 1, 2004 – December 31, 2014.

**Breast Cancer***(Caribbean OR West Indies OR Leeward OR Windward OR Antilles OR Anguilla OR Antigua OR Aruba OR Barbuda OR Bahamas OR Barbados OR Barthelemy OR “St. Bartholomew” OR “Saint Bartholomew” OR Barts OR Belize OR Bermuda OR Bonaire OR Cayman OR Croix OR Cuba OR Curacao OR Dominica OR “Dominican Republic” OR Eustatius OR “Santo Domingo” OR “Saint Domingue” OR “St. Domingue” OR Grenada OR Guadeloupe OR Guyana OR Haiti OR Hispaniola OR Jamaica OR “St. John” OR “Saint John” OR “St. Thomas” OR “Saint Thomas” OR “St. Vincent” OR “Saint Vincent” OR “St. Martin” OR “Saint Martin” OR “St. Maarten” OR “Saint Maarten” OR Martinique[tw] OR Martinique[AD] OR Martinique [TA] OR Martinique [LID] OR Martinique [PL] OR Martinique [PUBN] OR “St. Nevis” OR “Saint Nevis” OR “St. Christopher and Nevis” OR “Saint Christopher and Nevis” OR “St. Lucia” OR “Saint Lucia” OR Kitts OR Montserrat OR “Puerto Rico” OR Grenadines OR “Virgin Islands” OR Saba OR Suriname OR Trinidad OR Tobago OR Tortola) AND (age OR gender OR education OR educat*OR income OR wealth OR ethnic OR ethnic* OR race OR culture OR language OR occupation OR religion OR social class OR socioeconomic OR health social determinants OR social determinant* OR social capital OR residence OR medical geography OR health service OR health service* OR health equity OR disparit* OR medical sociology OR prejudice OR health insurance OR health gradient OR health gap OR vulnerable populations OR continental population groups OR Arawak* OR Amerindian* OR carib OR caribs OR taino* OR ethnic groups OR social conditions OR urban OR rural OR urban health OR urban population OR rural health OR rural population OR social position OR poverty OR wealth OR rich[tw] OR poor OR social support OR discriminat* OR differenti* OR globaliz* OR globalis* OR urbanization OR urbaniz* OR urbanis* OR westerniz* OR westernis*) AND (“Breast Neoplasms” [MeSH] OR ((breast OR ductal OR lobular OR (mammary AND gland)) AND (cancer OR sarcoma OR carcinoma OR tumor OR neoplasm OR mucosa associated lymphoid tissue OR MALT OR stage OR stages OR staging OR grade OR grades OR grading OR recurren* OR remission OR (year AND survival))) OR (Alcohol OR drinking or Bmi OR body mass index OR overweight OR obesity OR obes* OR sugar OR sweet* OR (Physical* AND (active* OR activi* OR inactiv*)) OR motor activity OR exercise OR radiation OR x-ray OR (age AND first AND (pregnancy OR pregnant OR birth)) OR maternal age OR Parity OR parous OR (Number AND ( children OR live births OR babies OR delivered OR deliveries)) OR breastfeed OR breastfeeding OR breastfed))*

**Prostate Cancer** *(Caribbean OR West Indies OR Leeward OR Windward OR Antilles OR Anguilla OR Antigua OR Aruba OR Barbuda OR Bahamas OR Barbados OR Barthelemy OR “St. Bartholomew” OR “Saint Bartholomew” OR Barts OR Belize OR Bermuda OR Bonaire OR Cayman OR Croix OR Cuba OR Curacao OR Dominica OR “Dominican Republic” OR Eustatius OR “Santo Domingo” OR “Saint Domingue” OR “St. Domingue” OR Grenada OR Guadeloupe OR Guyana OR Haiti OR Hispaniola OR Jamaica OR “St. John” OR “Saint John” OR “St. Thomas” OR “Saint Thomas” OR “St. Vincent” OR “Saint Vincent” OR “St. Martin” OR “Saint Martin” OR “St. Maarten” OR “Saint Maarten” OR Martinique[tw] OR Martinique[AD] OR Martinique [TA] OR Martinique [LID] OR Martinique [PL] OR Martinique [PUBN] OR “St. Nevis” OR “Saint Nevis” OR “St. Christopher and Nevis” OR “Saint Christopher and Nevis” OR “St. Lucia” OR “Saint Lucia” OR Kitts OR Montserrat OR “Puerto Rico” OR Grenadines OR “Virgin Islands” OR Saba OR Suriname OR Trinidad OR Tobago OR Tortola) AND (age OR gender OR education OR educat*OR income OR wealth OR ethnic OR ethnic* OR race OR culture OR language OR occupation OR religion OR social class OR socioeconomic OR health social determinants OR social determinant* OR social capital OR residence OR medical geography OR health service OR health service* OR health equity OR disparit* OR medical sociology OR prejudice OR health insurance OR health gradient OR health gap OR vulnerable populations OR continental population groups OR Arawak* OR Amerindian* OR carib OR caribs OR taino* OR ethnic groups OR social conditions OR urban OR rural OR urban health OR urban population OR rural health OR rural population OR social position OR poverty OR wealth OR rich[tw] OR poor OR social support OR discriminat* OR differenti* OR globaliz* OR globalis* OR urbanization OR urbaniz* OR urbanis* OR westerniz* OR westernis*) AND (“Prostate neoplasms” [MeSH] ((prostate OR prostatic) AND (cancer OR sarcoma OR carcinoma OR tumor OR neoplasm OR mucosa associated lymphoid tissue OR MALT OR stage OR stages OR staging OR grade OR grades OR grading OR recurren* OR remission OR (year AND survival))) OR (calcium OR milk OR dairy))*

## Appendix B – Redcap Data Extraction Forms


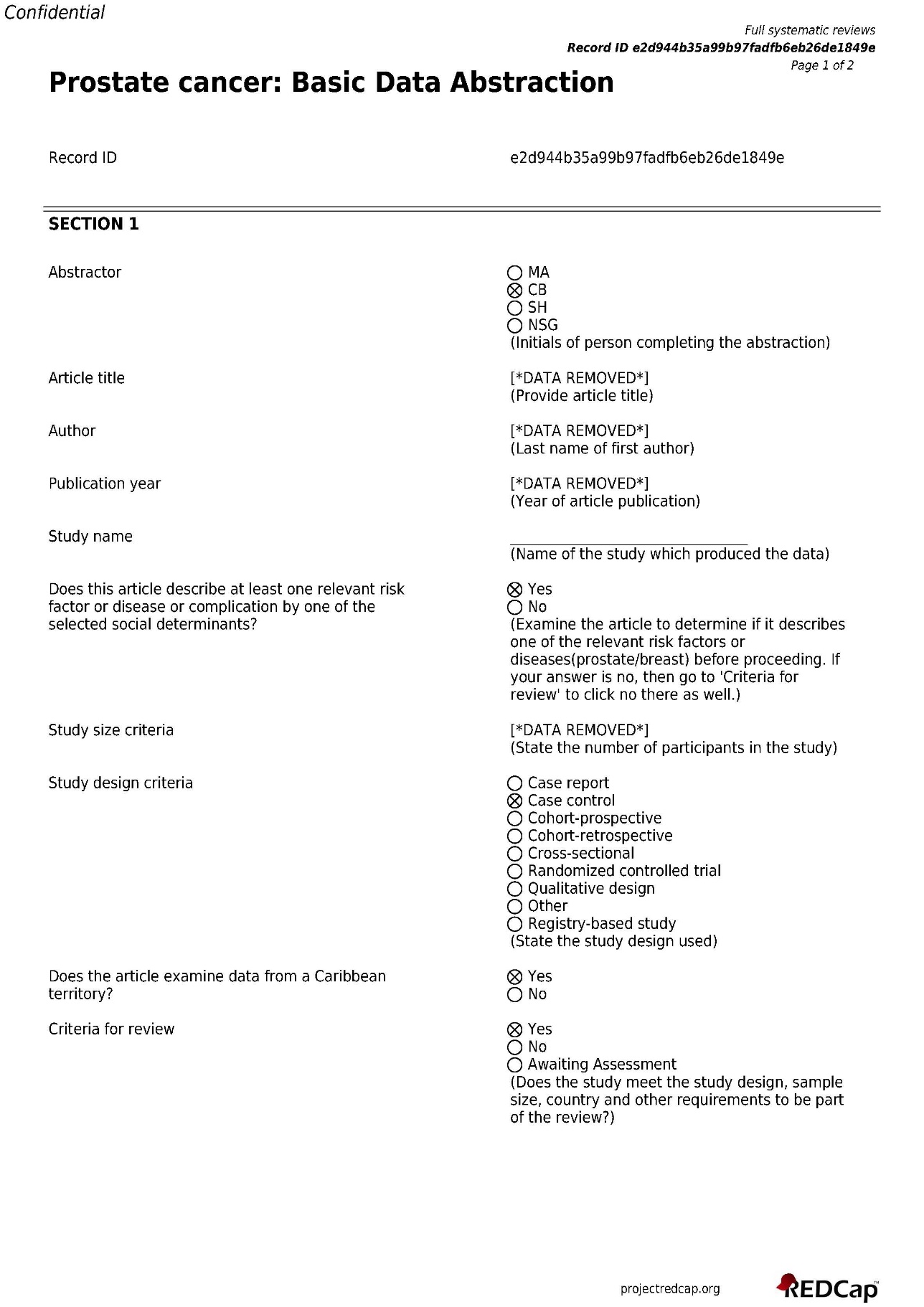


##
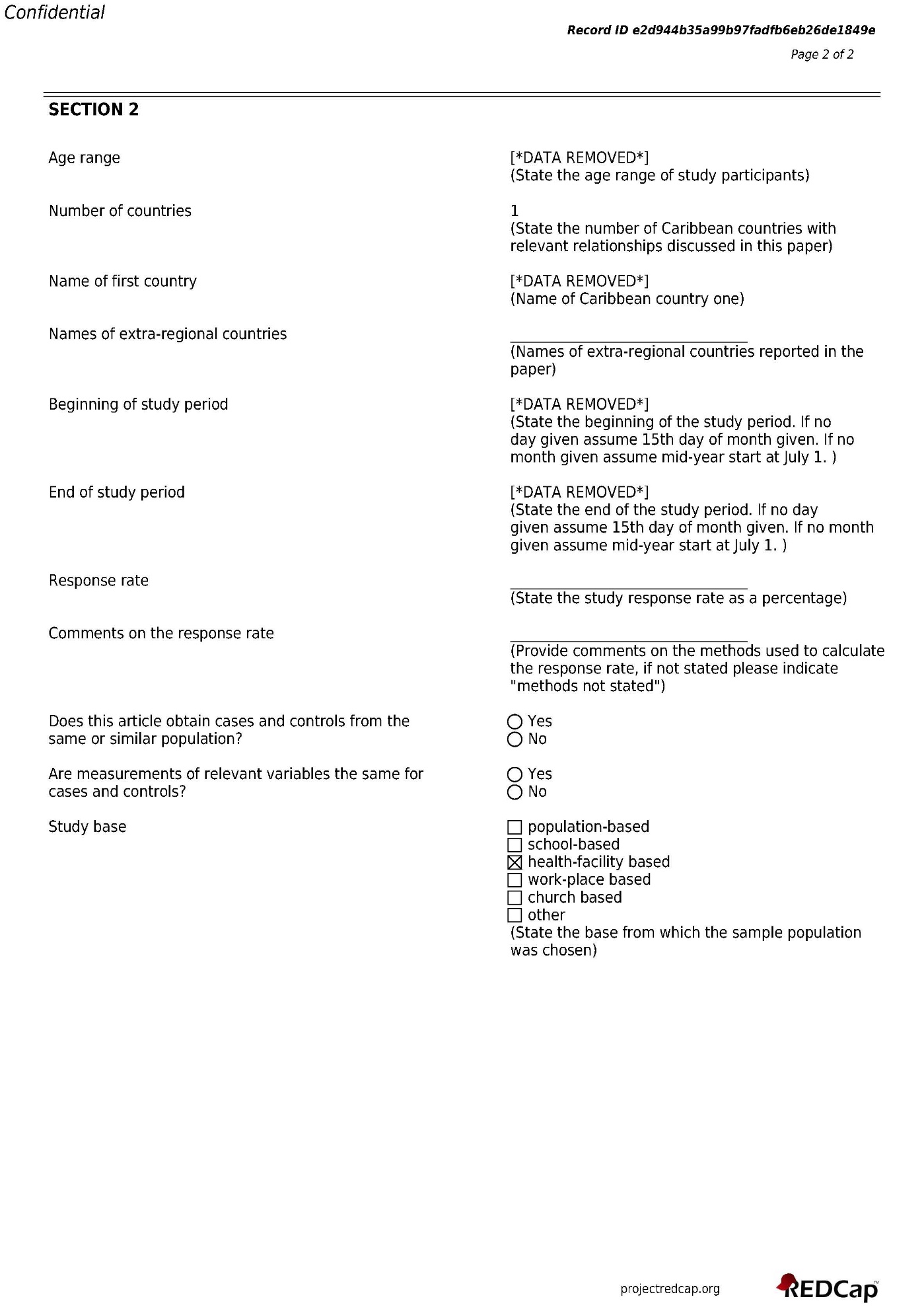

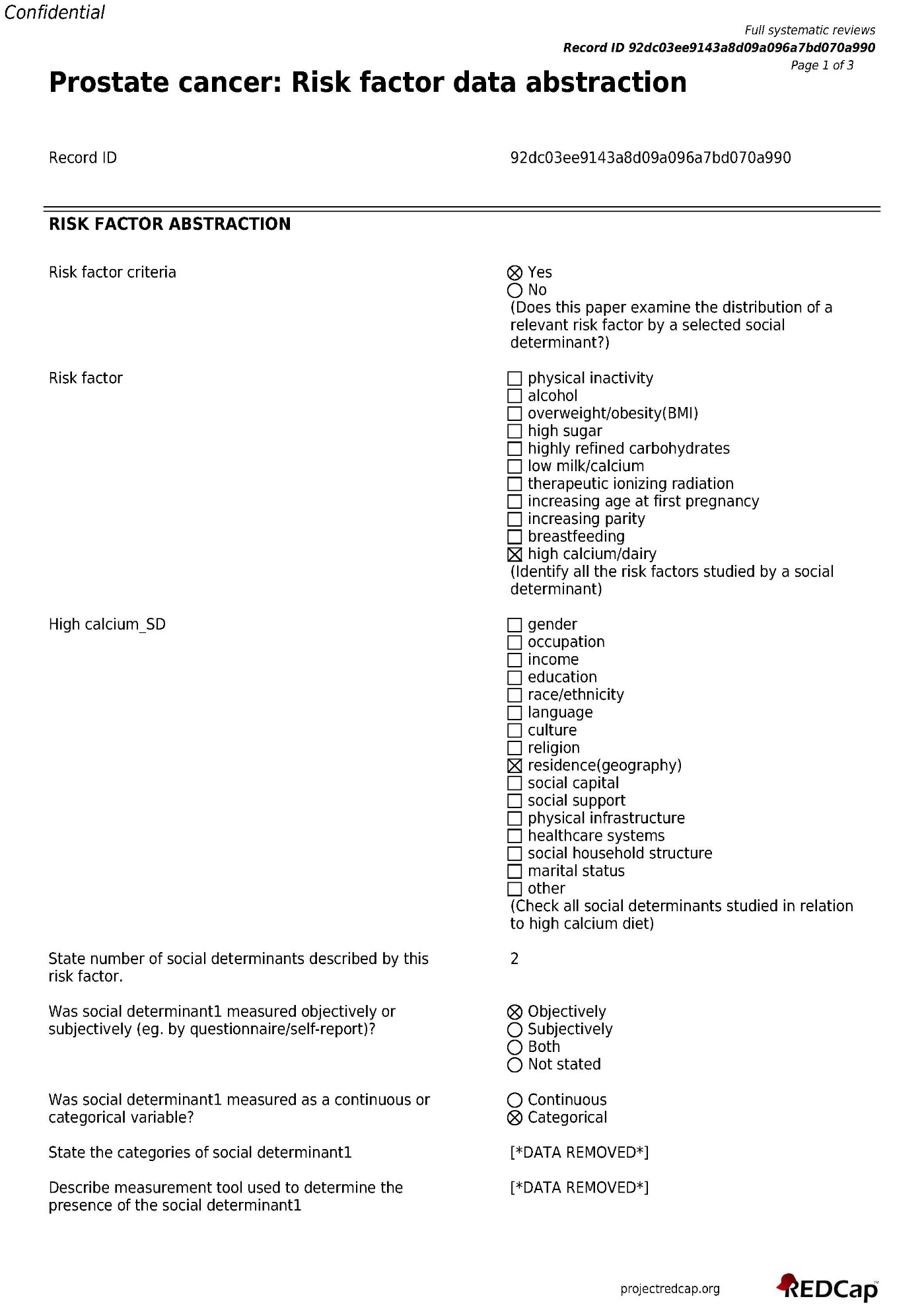

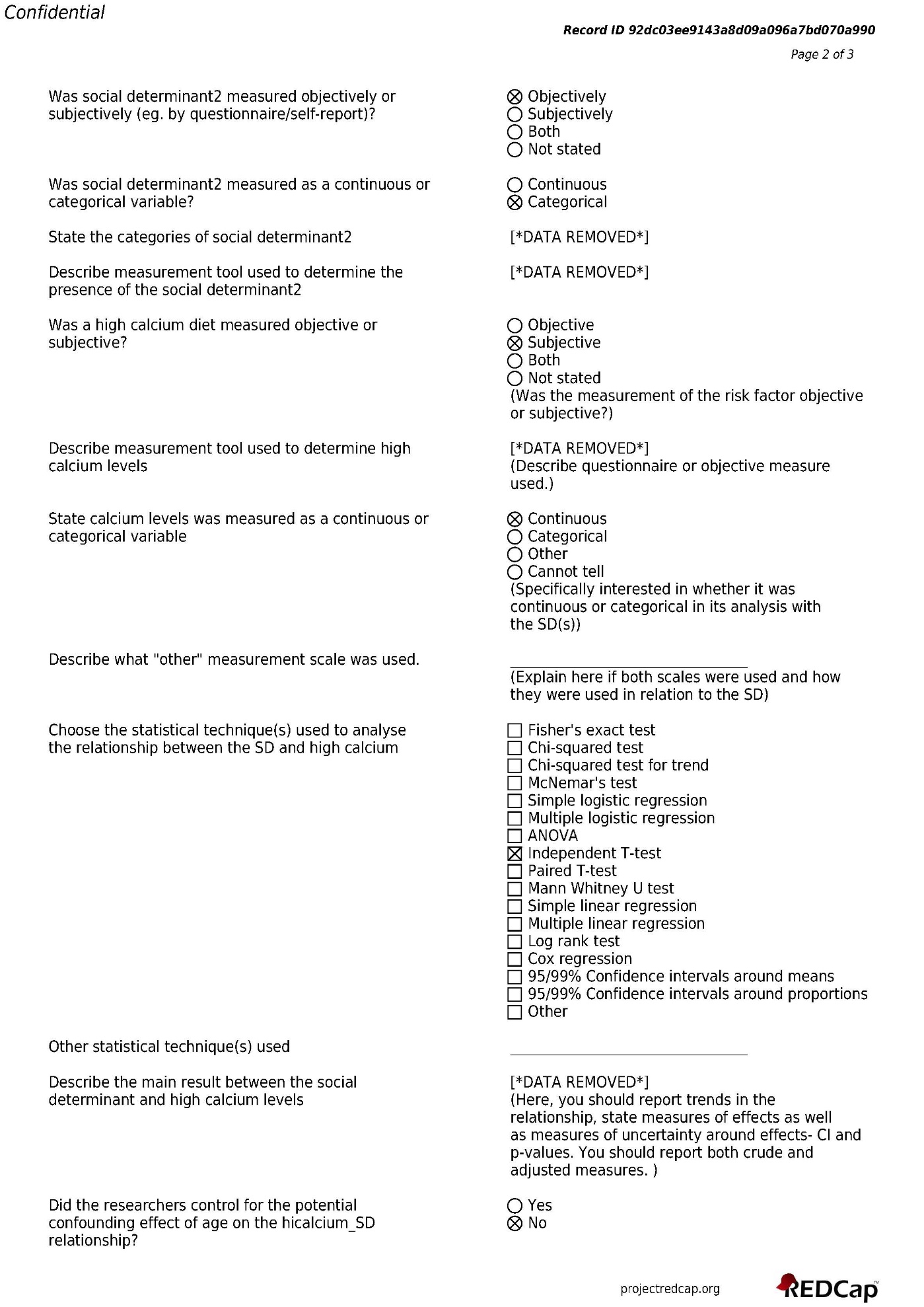

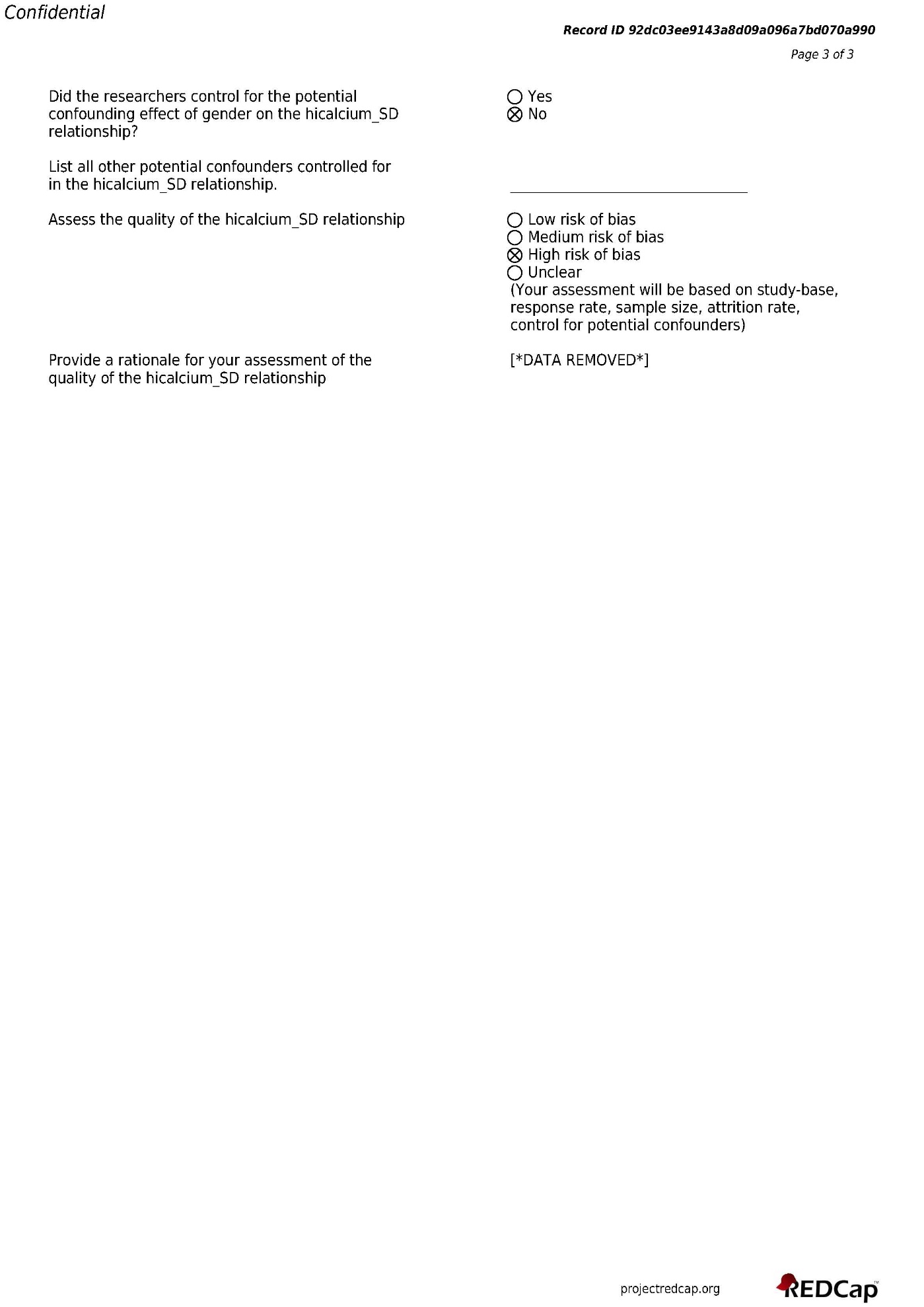


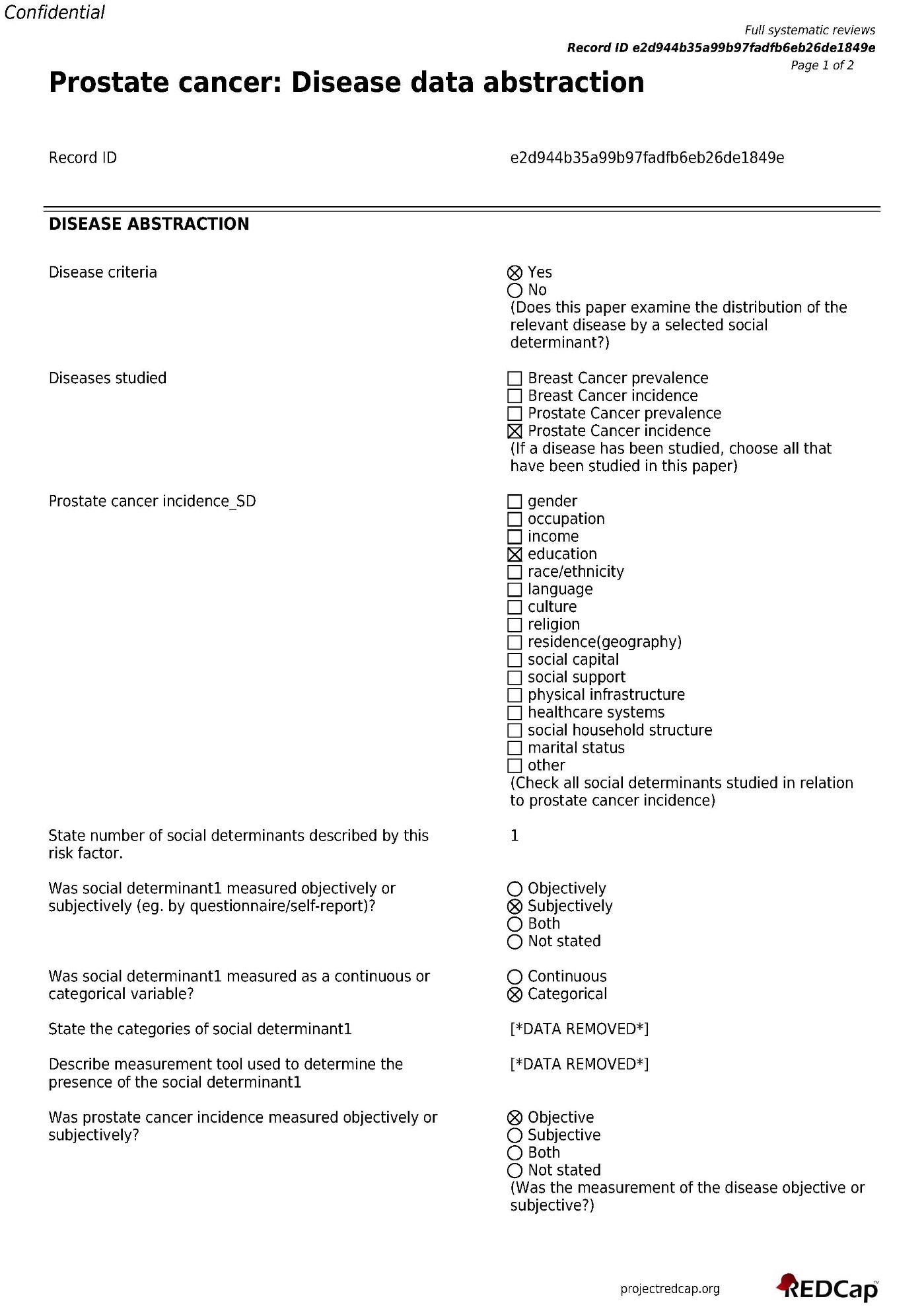


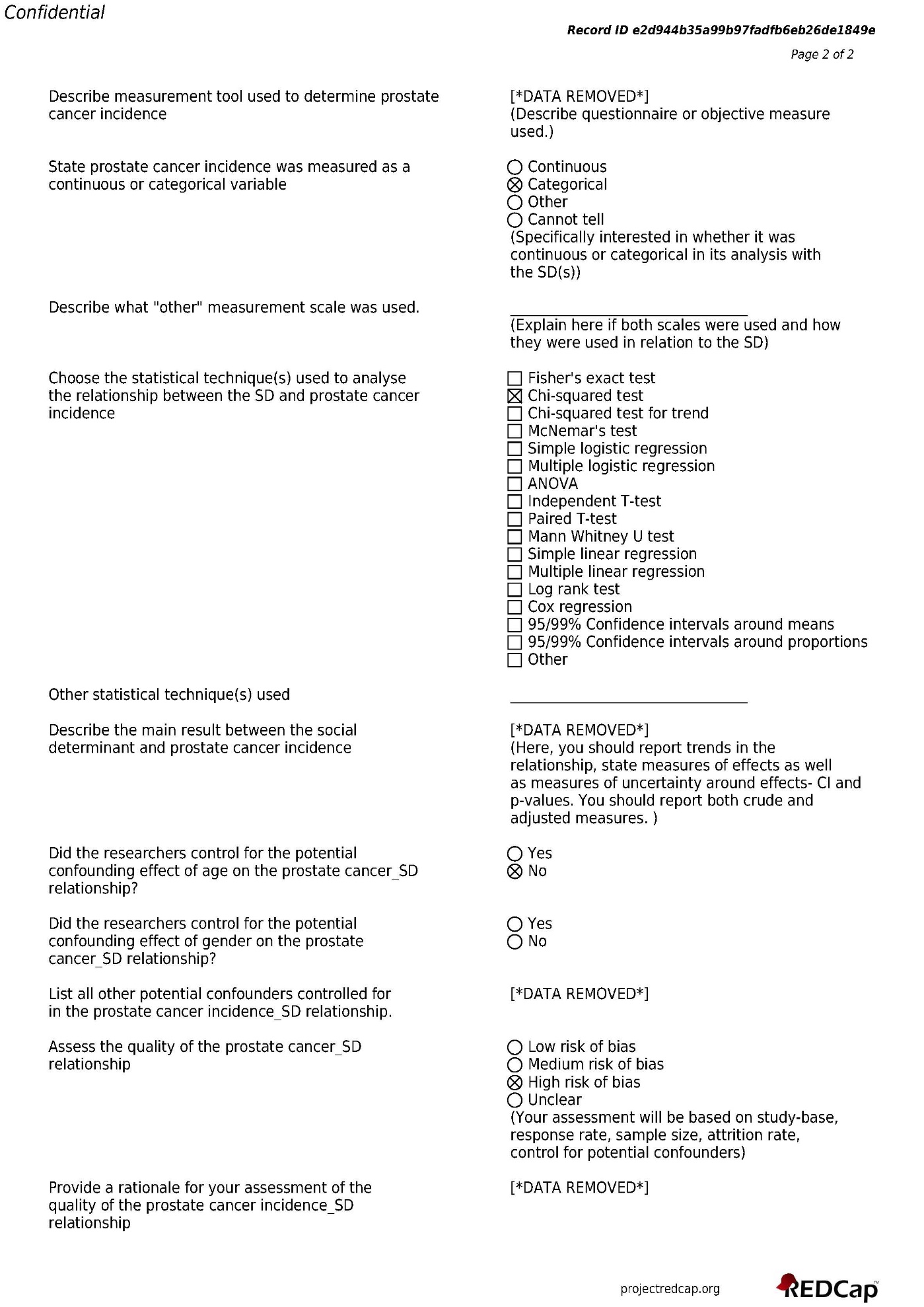


##
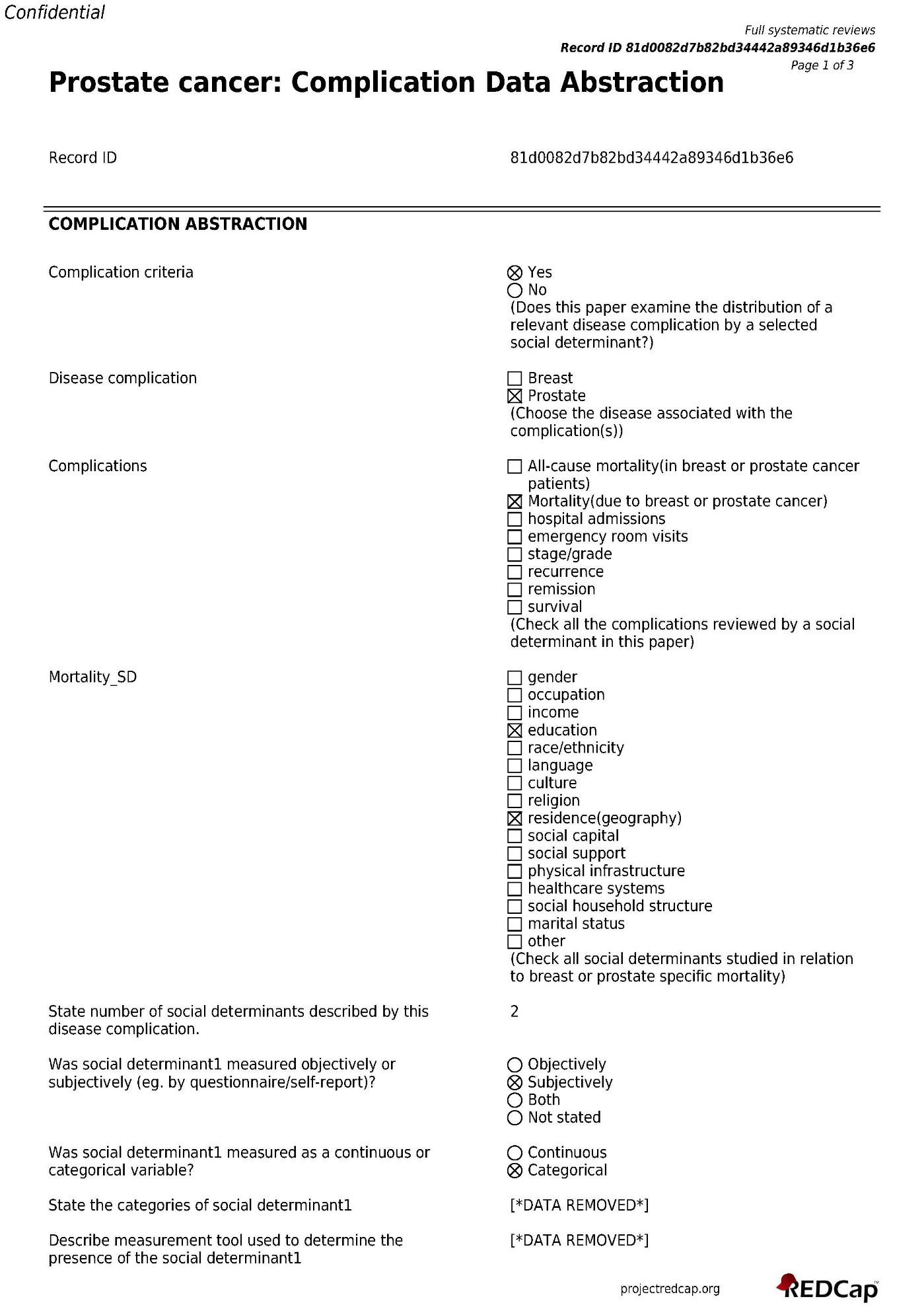

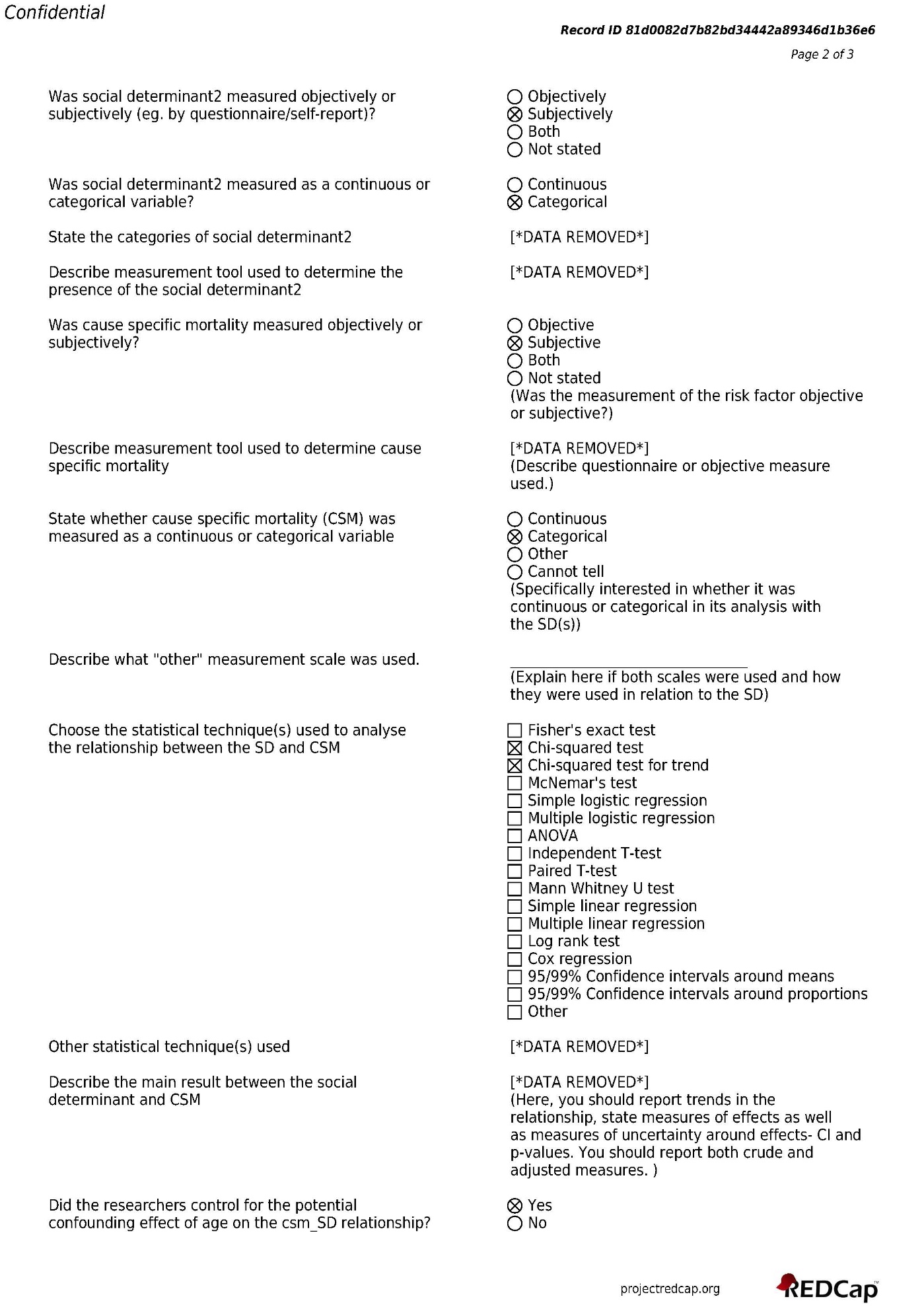

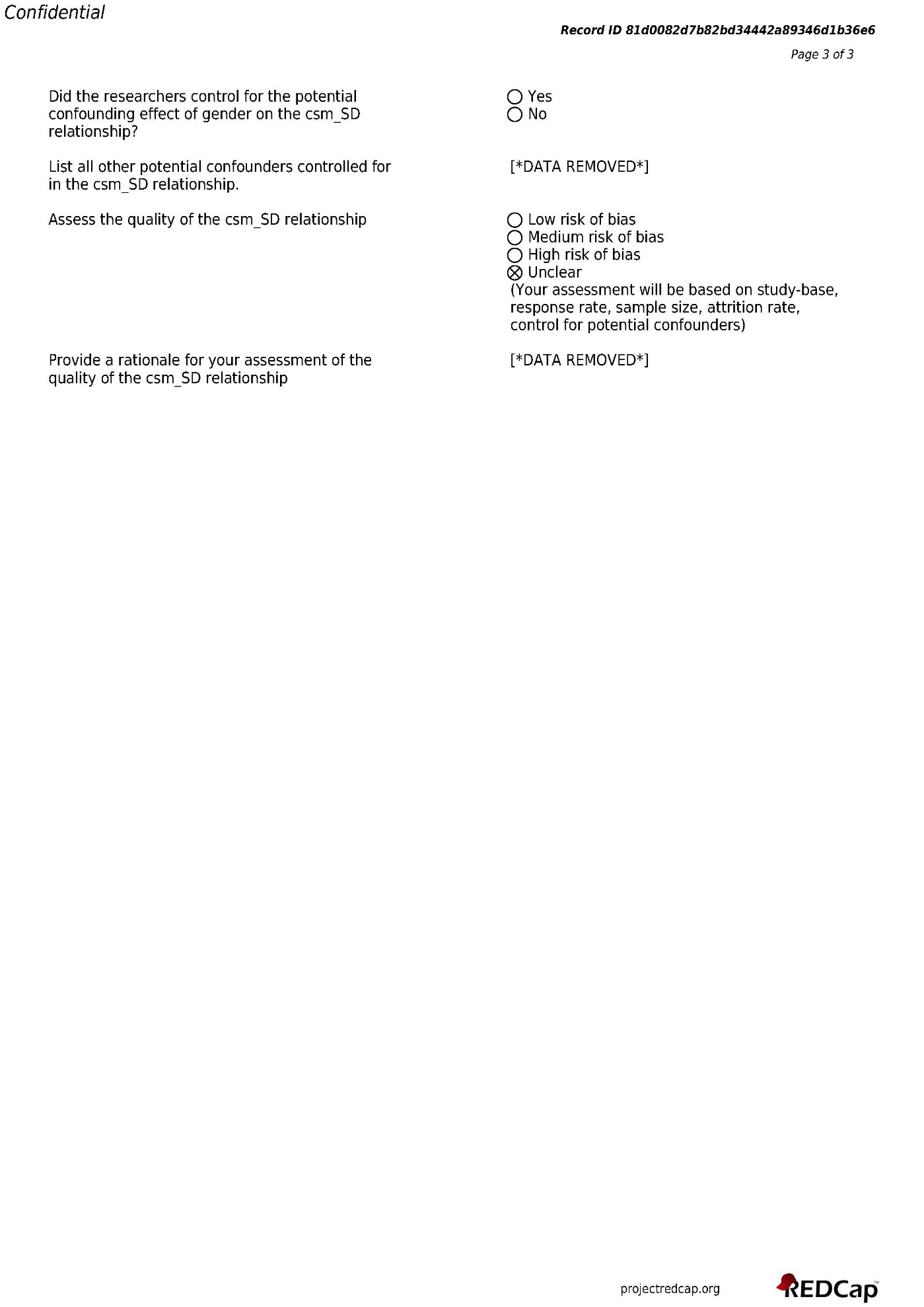
Appendix C – Risk of Bias Assessment Tool

Version 3.1

12-Dec-2015

This tool is a simplification of the Cochrane ACROBAT-NRSI tool, with adaptations to account for the fact that our systematic reviews do not include non-randomised studies of interventions (NRSI). The types of non-randomised studies that are assessed using this adapted tool are observational studies of any design that report relationships between a social determinant and known risk factors for a specific disease, disease frequency (such as incidence or prevalence), or disease outcomes (such as survival or mortality).

ACROBAT-NRSI is based on the Cochrane Risk of Bias (RoB) tool for randomized trials, which was launched in 2008 and modified in 2011. As in the tool for randomized trials, risk of bias is assessed within specified bias domains, and review authors document the information on which judgements are based.

The focus of this RoB tool is on internal validity. We define bias as a tendency for study results to differ systematically from the results expected from a study of the same design, conducted on the same participant group, and that had no flaws in its conduct. Such bias is distinct from issues of generalizability (applicability) to types of individual who were not included from the study.

The domains of bias used in this adapted RoB tool have the following meaning:

| Bias due to confounding | A confounding variable is a prognostic factor that may partly predict whether a participant has a particular value of a social determinant.  **Example.** For the relationship between level of education (a social determinant) and prostate cancer prevalence (a measure of disease frequency), age and sex would be important confounding factors as both age and sex would also be expected to influence a person's level of education. |
| --- | --- |
| Bias in participant selection | Selection bias occurs when some eligible participants are excluded in a way that leads to the association between the social determinant and the outcome differing from the association that might have been observed in the absence of these exclusions.  **Example.** For the relationship between level of education (a social determinant) and prostate cancer (a measure of disease frequency), participant non-selection may have been related to level of education, with (for example) those with lower levels of education less likely to participant in the study. |
| Bias due to missing data | Missing data may arise, among other reasons, through attrition (loss to follow up), missed appointments, incomplete data collection and by participants being excluded from analysis by primary investigators. In NRS, data may be missing for social determinants, for disease risk factors, frequency or outcomes, or for other variables involved in the analysis or a combination of these.  A general rule for consideration of bias due to missing data is that we should assume that an analysis using the data we intended to collect (were they available) would produce an unbiased effect estimate, so that biases might reasonably be introduced by any missing data. |
| Bias in measurement of outcomes | Bias may be introduced if social determinants, disease risk factors, disease frequency, or disease outcomes are misclassified or measured with error. |
| Bias in selection of reported results | Selective reporting is the failure to report, or partial reporting of relationships between social determinants and either risk factors, disease frequency, or disease outcomes that were measured and analysed. Selective reporting might be (a) selective reporting of a particular outcome measurement from multiple measurements; (b) selective reporting of a particular analysis from multiple analyses of a specific outcome measurement; and (c) selective reporting of a subset of the participants. |

**DOMAIN 1: Confounding.**

**Table A. Questions for each relationship
(one table to be completed for each relationship)**

| **Relationship Description:** |  |  |  |  |  |
| --- | --- | --- | --- | --- | --- |
|  | No | Possibly No | Possibly Yes | Yes | No Info |
| 1.1 Is confounding of the relationship between the social determinant and the disease endpoint unlikely in this study? |  |  |  |  |  |
| 1.2. Did the authors use an appropriate analysis method that adjusted for all the critically important confounding domains? |  |  |  |  |  |
| 1.3. Were confounders that were adjusted for measured validly and reliably by the variables available in this study? |  |  |  |  |  |

**Table B. How to Judge and Apply Risk of Bias to each relationship**

| Low Risk of Bias | No confounding expected |
| --- | --- |
| Moderate Risk of bias | Confounding expected, all known critically important confounding domains appropriately measured and adjusted for; **AND** Reliability and validity of measurement of critically important domains were sufficient that we do not expect serious residual confounding. |
| Serious Risk of Bias | At least one known critically important domain not appropriately measured, or not adjusted for; **OR** Reliability or validity of measurement of a critically important domain was low enough that we expect serious residual confounding. |
| Unclear Risk of Bias | No information on whether confounding might be present. |

**Table C. Risk of Bias Judgement for each relationship
(Add rows for >5 relationships)**

| **Relationship** | **Confounding** | **Comment** |
| --- | --- | --- |
| R1 |  |  |
| R2 |  |  |
| R3 |  |  |
| R4 |  |  |
| R5 |  |  |

**DOMAIN 2: Bias in Selection of Participants to Study.**

**Table A1. Cross-sectional & Registry Studies - questions for each relationship
(one table to be completed for each relationship)**

| **Relationship Description:** |  |  |  |  | |
| --- | --- | --- | --- | --- | --- |
|  | No | Possibly No | Possibly Yes | Yes | No Info |
| 2.1 Representative of target population * |  |  |  |  |  |
|  | <50% | 50 to 75% | 75% to 90% | >90% | No Info |
| 2.2 Response rate ** |  |  |  |  |  |

*** Target population need not be general population. Also, registry-based studies will be examined as cross-sectional studies; the quality of the registry will be assessed via Question 2.1 only.
** Not applicable to registry-based studies.**

**Table A2. Cohort Studies - questions for each relationship
(one table to be completed for each relationship)**

| **Relationship Description:** |  |  |  |  |  |
| --- | --- | --- | --- | --- | --- |
|  | No | Possibly No | Possibly Yes | Yes | No Info |
| 2.3 Representative of target population * |  |  |  |  |  |
|  | >50% | 25% to 50% | 10% to 25% | <10% | No Info |
| 2.4 Attrition rate |  |  |  |  |  |

*** Target population need not be general population.**

**Table A3. Case-Control Studies - questions for each relationship
(one table to be completed for each relationship)**

| **Relationship Description:** |  |  |  |  |  |
| --- | --- | --- | --- | --- | --- |
|  | No | Possibly No | Possibly Yes | Yes | No Info |
| 2.5 Cases and Controls taken from same or similar population |  |  |  |  |  |

**Table B. How to Judge and Apply Risk of Bias to each relationship**

| Low Risk of Bias | **Cross-sectional:** Representative of target population **AND** response rate >75%  **Cohort:** Representative of target population **AND** Attrition rate <10%  **Case-Control:** Cases and controls from same or similar populations |
| --- | --- |
| Moderate Risk of bias | **Cross-sectional:** Representative of target population **AND** response rate 50%-75%  **Cohort:** Representative of target population **AND** Attrition rate 25-50%  **Case-Control:** Cases and controls possibly from same or similar populations |
| Serious Risk of Bias | **Cross-sectional:** Not representative of target population **OR** response rate <50%  **Cohort:** Not representative of target population **OR** Attrition rate >50%  **Case-Control:** Cases and controls possibly not or not from same or similar populations |
| Unclear Risk of Bias | No information on whether confounding might be present. |

**Table C. Risk of Bias Judgement for each relationship
(Add rows for >5 relationships)**

| **Relationship** | **Selection of Participants** | **Comment** |
| --- | --- | --- |
| R1 |  |  |
| R2 |  |  |
| R3 |  |  |
| R4 |  |  |
| R5 |  |  |

**DOMAIN 3: Bias due to missing data.**

**Table A. Questions for each relationship
(one table to be completed for each relationship)**

| **Relationship Description:** |  |  |  |  |  |
| --- | --- | --- | --- | --- | --- |
|  | >20% | 15% to 20% | 10% to 15% | <10% | No Info |
| 3.1 Exclusion of potentially eligible participants because of missing data |  |  |  |  |  |
|  | No | Possibly No | Possibly Yes | Yes | No Info |
| 3.2 Were appropriate statistical methods used to account for missing data |  |  |  |  |  |

**Table B. How to Judge and Apply Risk of Bias to each relationship**

| Low Risk of Bias | Data were reasonably complete (<10% missing) **OR** appropriate statistical analyses used to account for missing data |
| --- | --- |
| Moderate Risk of bias | Missing data (10-20%) **AND** missing data not addressed in the analysis |
| Serious Risk of Bias | Missing data (>20%) regardless if addressed in the analysis. |
| Unclear Risk of Bias | No information on whether confounding might be present. |

**Table C. Risk of Bias Judgement for each relationship
(Add rows for >5 relationships)**

| **Relationship** | **Missing  Data** | **Comment** |
| --- | --- | --- |
| R1 |  |  |
| R2 |  |  |
| R3 |  |  |
| R4 |  |  |
| R5 |  |  |

**DOMAIN 4: Bias in measurement of outcomes.**

**Table A. Questions for each relationship
(one table to be completed for each relationship)**

| **Relationship Description:** |  |  |  |  |  |
| --- | --- | --- | --- | --- | --- |
|  | No | Possibly No | Possibly Yes | Yes | No Info |
| 4.1 Social determinant is appropriately measured? * |  |  |  |  |  |
| 4.2 Risk factor / disease frequency / disease outcome measured objectively ** |  |  |  |  |  |

*** Social determinants measured via self-report would likely be listed as “Possibly Yes”
** Risk factors which are unlikely to be measured objectively (alcohol, physical activity), and are instead measured via self-report, can be considered as “possibly yes”**

**Table B. How to Judge and Apply Risk of Bias to each relationship**

| Low Risk of Bias * | Social determinant is appropriately measured (yes or possibly yes) **AND** risk factor / disease frequency / disease outcome is objectively measured (yes / possibly yes) |
| --- | --- |
| Moderate Risk of bias | Social determinant **not** appropriately measured (no or possibly no) **AND** Risk factor / disease frequency / disease outcome is objectively measured (yes / possibly yes) |
| Serious Risk of Bias | Social determinant **not** appropriately measured (no or possibly no) **AND** risk factor / disease frequency / disease outcome **not** objectively measured (no or possibly no) |
| Unclear Risk of Bias | No information on whether confounding might be present. |

*** EXCEPTION: If social determinant and risk factor are measured via self-report out of necessity (eg: alcohol consumption), then risk of bias is considered as moderate, not low.**

**Table C. Risk of Bias Judgement for each relationship
(Add rows for >5 relationships)**

| **Relationship** | **Measurement  of Outcomes** | **Comment** |
| --- | --- | --- |
| R1 |  |  |
| R2 |  |  |
| R3 |  |  |
| R4 |  |  |
| R5 |  |  |

**DOMAIN 5: Bias in selection of the reported results.**

**Table A. Questions for each relationship
(one table to be completed for each relationship)**

| **Relationship Description:** |  |  |  |  |  |
| --- | --- | --- | --- | --- | --- |
|  | No | Possibly No | Possibly Yes | Yes | No Info |
| 5.1 From the study report, do the results section and figures/tables reflect the data and analyses described in the study methods * |  |  |  |  |  |
| 5.2 Is there evidence of multiple endpoints within the same endpoint domain ** |  |  |  |  |  |
| 5.3 Is there evidence of multiple analyses for a single social determinant-endpoint relationship *** |  |  |  |  |  |

**“* If paper describes the methods section poorly, this would likely be listed as “No Info”
** An example of this might be BMI and Waist Circumference, both used as measures of adiposity. Also, this does not refer to the abstractors’ own constructs (eg: if article lists maternal age, maternal education as single independent variables, and abstractor categorizes all as proxies of SES)
*** This question relates directly to 5.2 only, referring to multiple analyses of a single endpoint domain with multiple endpoints examined differently. An example of this might be univariate, then adjusted analyses for the same relationship.**

**Table B. How to Judge and Apply Risk of Bias to each relationship**

| Low Risk of Bias | There is clear evidence (through examination of a protocol or statistical analysis plan) that all reported results correspond to all intended outcomes and analyses. |
| --- | --- |
| Moderate Risk of Bias | Relationship and analyses are not consistent with a stated a priori plan, but there is no absolute evidence of selective endpoint use or of multiple analyses for the same relationship. |
| Serious Risk of Bias | Relationship and analyses are not consistent with an a priori plan **OR** there is absolute evidence (“Yes” answers only) of selective endpoint use **OR** of multiple analyses for the same relationship |
| Unclear Risk of Bias | No information on whether confounding might be present. |

**Table C. Risk of Bias Judgement for each relationship
(Add rows for >5 relationships)**

| **Relationship** | **Selected**  **Reporting** | **Comment** |
| --- | --- | --- |
| R1 |  |  |
| R2 |  |  |
| R3 |  |  |
| R4 |  |  |
| R5 |  |  |

**ALL DOMAINS: Summary Risk of Bias Table.**

**Risk of Bias Judgement for all domains combined (Add rows for >5 relationships)**

No definitive criteria for determining the Overall RoB as is subjectively based on the qualitative feel of the paper. A general rule of thumb might be that the Overall RoB is most likely to be the same as the worst classification of 5 Domains, but with exceptions.

| **Relationship** | **Domain 1: Confounding** | **Domain 2: Selection of Participants** | **Domain 3: Missing  Data** | **Domain 4: Measurement  of Outcomes** | **Domain 5: Selected**  **Reporting** | **OVERALL RoB** |
| --- | --- | --- | --- | --- | --- | --- |
| **R1** |  |  |  |  |  |  |
| **R2** |  |  |  |  |  |  |
| **R3** |  |  |  |  |  |  |
| **R4** |  |  |  |  |  |  |
| **R5** |  |  |  |  |  |  |
